# Bone‐Derived dECM Hydrogels Support Tunable Microenvironments for In Vitro Osteogenic Differentiation

**DOI:** 10.1002/adhm.202501350

**Published:** 2025-10-02

**Authors:** Minne Dekker, Luke Hipwood, Akhilandeshwari Ravichandran, Dietmar W. Hutmacher, Christoph Meinert, Jacqui McGovern

**Affiliations:** ^1^ Faculty of Health School of Biomedical Sciences Queensland University of Technology (QUT) Brisbane 4000 Australia; ^2^ School of Mechanical Medical and Process Engineering Faculty of Engineering Queensland University of Technology Brisbane 4000 Australia; ^3^ Centre for Biomedical Technologies Queensland University of Technology Brisbane 4059 Australia; ^4^ Gelomics Pty Ltd Brisbane 4059 Australia; ^5^ Max Planck Queensland Centre (MPQC) for the Materials Science of Extracellular Matrices Queensland University of Technology Brisbane 4059 Australia; ^6^ Australian Research Council (ARC) Training Centre for Multiscale 3D Imaging Modelling and Manufacturing (M3D Innovation) Queensland University of Technology Brisbane 4000 Australia; ^7^ Translational Research Institute QUT Woolloongabba 4102 Australia; ^8^ ARC Training Centre for Cell and Tissue Engineering Technologies (CTET) QUT Brisbane 4000 Australia; ^9^ Queensland University of Technology 60 Musk Ave Kelvin Grove Queensland 4059 Australia; ^10^ Gelomics Pty Ltd 60 Musk Ave Kelvin Grove Queensland 4059 Australia; ^11^ Translational Research Institute 37 Kent St Woolloongabba Queensland 4102 Australia

**Keywords:** bone‐derived extracellular matrices (dECM), biomimetic hydrogels, osteogenic differentiation, photocrosslinkable hydrogels, tunable mechanical properties

## Abstract

Decellularized extracellular matrix (dECM)‐based biomaterials mimic native ECM and support 3D cell culture. A photocrosslinkable porcine bone‐derived dECM hydrogel (dECM‐MA) is developed with tunable mechanical properties for tissue‐specific in vitro models. Trabecular bone is demineralized with 10% EDTA and decellularized via osmotic shock using 3.4 m NaCl, reducing DNA content by 94% while preserving key ECM proteins. Proteomic analysis identifies 81 matrisome proteins, with 76 shared between native and decellularized tissue. The dECM is solubilized by pepsin digestion and functionalized with methacryloyl groups, achieving 87–98% functionalization. Photocrosslinked dECM‐MA hydrogels shows tunable Young's moduli (0.5–120 kPa) depending on polymer concentration (0.25–2% w/v) and crosslinking duration (8–120 s). Primary human osteoblasts (hOBs) encapsulated in dECM‐MA (5, 10, and 20 kPa) remains viable and exhibits osteogenic morphology. In 10 kPa hydrogels, hOBs shows increased metabolic activity, elevated alkaline phosphatase, and mineral deposition (µCT, Alizarin Red). Expression of DMP‐1 and osteocalcin indicates cell maturation and ECM remodeling. This study demonstrates the feasibility of creating tunable, bone‐specific dECM hydrogels for 3D culture. dECM‐MA provides a controllable matrix environment and represents a versatile platform for disease modeling and drug screening in tissue‐specific microenvironments.

## Graphical abstract

A tunable methacrylated decellularized bone matrix hydrogel (dECM‐MA) is developed to support 3D culture of human osteoblasts. The hydrogel preserves bone‐specific ECM cues and allows precise control over mechanical properties. This system provides a customizable platform for studying osteogenic differentiation and modeling bone tissue environments for regenerative medicine and drug testing applications.

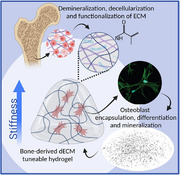

## 1 Introduction

The bone extracellular matrix (ECM) provides a highly specialized microenvironment that orchestrates cellular behavior through tightly regulated mechanical and biochemical cues. Bone regeneration relies on biomaterials that can mimic the native ECM to support osteogenic differentiation and facilitate tissue repair.^[^ ^1^ ^]^ Biomimetic hydrogels have gained significant attention for their ability to provide a 3D microenvironment that replicates key aspects of the ECM, enhancing cellular interactions and promoting functional tissue development.^[^ ^2^ ^]^ Bone ECM is composed of organic and inorganic components, with collagen type I making up 90% of the organic fraction, while the remaining 10% consists of non‐collagenous proteins such as glycoproteins and proteoglycans.^[^ ^1^ ^]^ The native bone ECM is a highly dynamic structure, orchestrating cellular responses through biochemical and mechanical cues, regulating remodeling and bone regeneration.^[^ ^1^ ^]^ This dynamic environment interacts with resident bone cells, including osteoblasts and osteoclasts, to regulate remodeling and regeneration.^[^ ^1^ ^]^

Bone defects caused by trauma, disease, or surgical resection present significant clinical challenges and are typically treated using autologous or allogeneic bone grafts, as well as synthetic bone substitutes. However, these traditional approaches are limited by donor tissue availability and the risk of immune rejection.^[^ ^3^ ^]^ Hydrogels have emerged as promising alternatives due to their biocompatibility, structural similarity to the ECM, and tunable physical and biochemical properties, making them suitable candidates for engineering bone‐like microenvironments.^[^ ^3^, ^4^ ^]^ However, conventional hydrogel systems—including natural (e.g., collagen, alginate), synthetic (e.g., polyethylene glycol), and semi‐synthetic (e.g., GelMA) polymers^[^ ^5^ ^]^—fail to fully replicate the biochemical complexity of bone ECM, limiting their effectiveness in both clinical and research applications.^[^ ^3^, ^6^ ^]^

Decellularized ECM (dECM) has emerged as a promising alternative, providing a naturally derived scaffold that better preserves the biochemical cues essential for bone tissue engineering.^[^ ^7^, ^8^ ^]^ Decellularization removes cellular components and immunogenic material while retaining ECM proteins, growth factors, and cytokines that regulate cell behavior and differentiation.^[^ ^7^ ^]^ The bone ECM contains growth factors and cytokines that can be maintained through the decellularization process, contributing to the regulation of cell behavior and differentiation.^[^ ^9^ ^]^ dECM enables the development of tissue‐specific hydrogels that closely replicate the biochemical properties of the native microenvironment, supporting cellular adhesion, proliferation, and differentiation. Bone‐derived ECM hydrogels have demonstrated superior bioactivity compared to conventional hydrogels, promoting osteogenic differentiation in multiple cell types, including C2C12 myoblasts and primary mouse calvarial cells.^[^ ^10^, ^11^ ^]^ Similarly, human bone‐derived ECM hydrogels have been shown to enhance osteogenic differentiation of human bone marrow stromal cells (hBMSCs) relative to gelatin‐based hydrogels.^[^ ^12^ ^]^

Despite these advantages, a major limitation of dECM hydrogels is the lack of precise control over key material properties such as stiffness, crosslink density, and degradation rate. These factors are critical in directing cell fate and optimizing biomaterial performance for specific applications. To address this, chemically functionalized ECM‐derived hydrogels have been developed, allowing for tunable physicochemical properties that enhance reproducibility and mechanical stability in tissue engineering applications.^[^ ^13^ ^]^ Functionalization of ECM‐based biomaterials enables fine‐tuning of mechanical properties, optimizing hydrogel stiffness to better support osteogenic differentiation. For example, endothelial cell vascularization has been demonstrated in functionalized bone‐derived ECM with adjustable mechanical properties, highlighting its regenerative potential.^[^ ^14^ ^]^

In osteogenic differentiation, hydrogel stiffness plays a critical role in regulating cell behavior. Studes have shown that the Young's modulus of GelMA hydrogels significantly influences osteoprogenitor differentiation, with softer hydrogels enhancing mineralization compared to stiffer formulations.^[^ ^14^ ^]^ These findings emphasize the necessity of designing biomaterials that integrate both the biochemical complexity of native bone ECM and precise mechanical control to regulate osteogenic differentiation. Apart form the hydrogel range of stiffness, other mechanical properties such as the porosity of a scaffold can influence the cell behaviour including cell migration, proliferation and viability.^[^ ^15^ ^]^ Studies have found that a higher porosity enhances osteogenisis.^[^ ^16^ ^]^ In this study, we developed a photocrosslinkable, bone‐derived ECM hydrogel with tunable mechanical properties suitable for osteogenic differentiation. Porcine trabecular bone demineralization and decellularization optimization, and ECM preservation were validated through DNA quantification, histological analysis, and proteomic characterization. The dECM was solubilized via pepsin digestion and functionalized with methacryloyl groups, enabling precise control of hydrogel stiffness. Primary human osteoblasts (hOBs) were encapsulated in dECM‐MA hydrogels and cultured for 8 weeks, demonstrating high viability, metabolic activity, and morphology changes consistent with osteogenic differentiation. Gene and protein expression analysis confirmed osteogenic marker expression across all stiffness conditions, including osteocalcin, collagen I, and dentin matrix protein‐1 (DMP‐1). However, only hOBs in dECM‐MA hydrogels with a Young's modulus of 10 kPa exhibited significant mineralization, as confirmed by microcomputed tomography (µCT) and IHC analysis.

## 2 Results

### 2.1 Optimization of Demineralization and Decellularization of Porcine Bone Tissue

**Figure 1:**
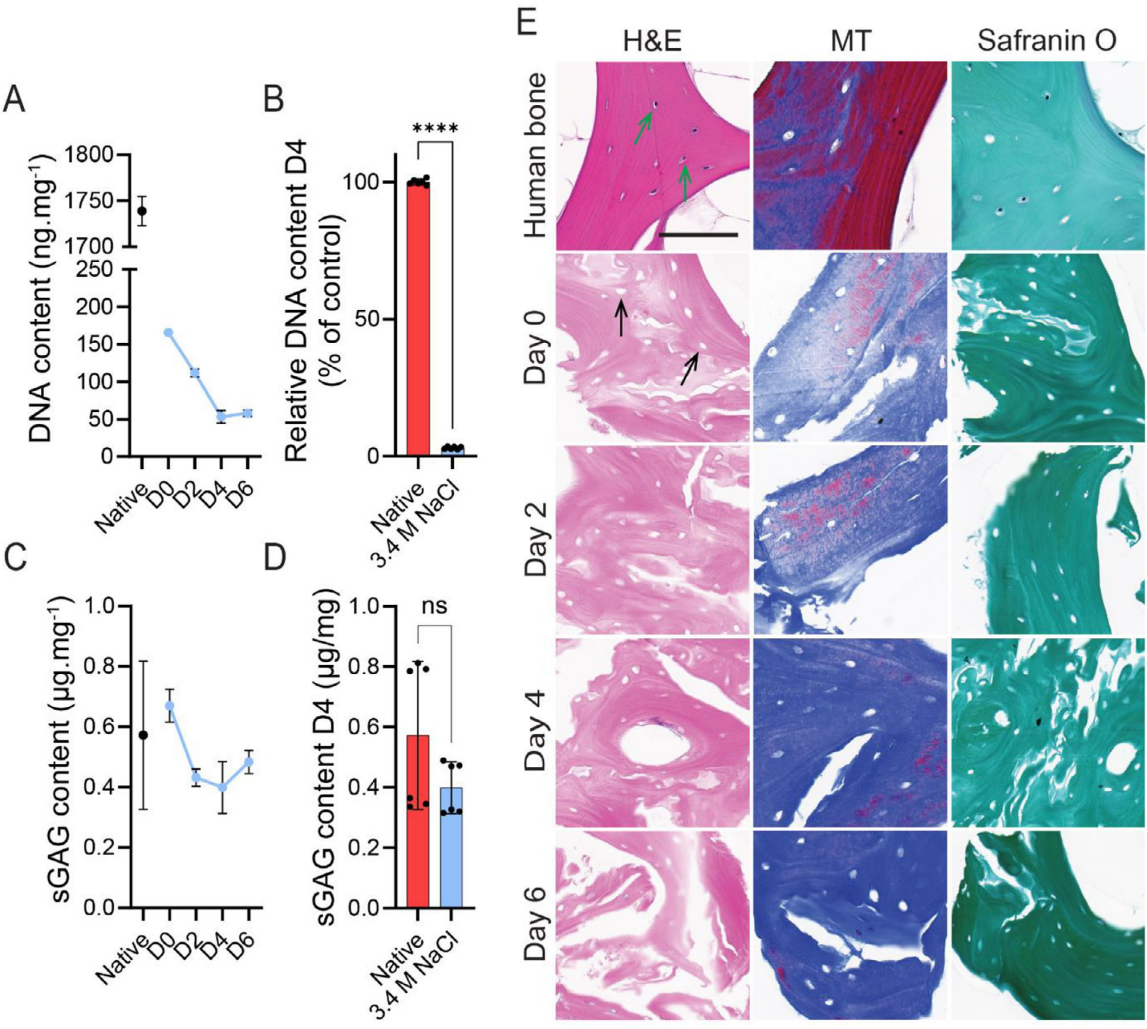
Demineralization and decellularization of porcine trabecular bone tissue. A) DNA content (ng.mg^−1^ wet tissue weight) of dECM decellularized with 3.4 m NaCl compared to native porcine bone tissue, and B) the percentage of DNA removal relative to native tissue on day 4 post‐demineralization. Statistical analysis was performed using an unpaired t test, with significant findings indicated by ^****^ *p* < 0.0001. C) Sulphated GAG content (µg.mg^−1^ wet tissue weight) of dECM decellularized with 3.4 m NaCl compared to native porcine bone tissue, and D) the Sulphated GAG content on day 4 post‐demineralization. No significant differences were observed when compared to the control, as determined by an unpaired t‐test. Data represent mean ± SD for 6 replicates (*n* = 2, 2 biological and 3 technical). E) Hematoxylin and Eosin staining (H&E), Masson's and Trichrome staining (MT), and Safranin O staining of 4 µm sections of the decellularized dECM on day 0 to day 6 compared to fixed and demineralized human bone tissue. The black arrows show the location of absent cells, while the green arrows show the location of cells indicated in purple present in the control. The scalebar = 100 µm, and the magnification is 40x.

Porcine trabecular bone was demineralized (Figure S1, Supporting Information) and decellularized to generate decellularized ECM (dECM) while preserving its biochemical integrity. To optimize decellularization protocols, four methods were compared over a 6‐day period: osmotic shock induced by 3.4 m NaCl, 1% sodium lauryl ether sulfate (SLES), 1% Triton X‐100, and a combination of 1% SLES and 1% Triton X‐100, with the latter involving 3 days in SLES followed by 3 days in Triton.^[^ ^17^, ^18^ ^]^ Osmotic shock (**Figure** **1**A) and SLES were significantly more effective at removing cellular content (Figure S2, Supporting Information); however, osmotic shock demonstrated superior cytocompatibility (Figure S3, Supporting Information) and was therefore selected as the preferred method for downstream analysis and bioassays.

DNA quantification confirmed progressive cell removal, with EDTA demineralization itself reducing DNA content significantly by day 0 compared to native bone (Figure 1A), even before treatment with NaCl. By day 4, the NaCl osmotic shock protocol had removed 94% of double‐stranded DNA (Figure 1B), consistent with previous studies.^[^ ^19^, ^20^, ^21^ ^]^ Histological analysis (H&E staining) further validated the absence of cell nuclei from day 0, immediately following demineralization, to day 6 (Figure 1E), with no significant differences observed in NaCl‐decellularized samples compared to other decellularization methods by day 4 and day 6 (Figure S2B, Supporting Information).

In addition to cell removal, preserving ECM proteins such as collagen, elastin, fibronectin, laminin, proteoglycan, and glycosaminoglycan (GAG) is critical to achieving biomimetic biochemical properties in dECM‐based hydrogels.^[^ ^1^, ^22^ ^]^ Sulfated GAG levels remained unchanged compared to native bone (Figure 1C,D), indicating successful ECM preservation, which was further confirmed using Safranin O staining (Figure 1E). Masson's Trichrome staining differentiates tissue based on density, with lower‐density regions appearing blue and higher‐density regions appearing red. The blue staining confirmed the presence of retained collagen fibrils, while the red areas indicated regions of higher tissue density, which may correspond to remaining mineralized components or more mature, compact bone.^[^ ^23^ ^]^

These findings establish osmotic shock as a highly effective, cytocompatible decellularization method, ensuring efficient DNA removal while maintaining ECM integrity, making it well‐suited for 3D cell culture and bone tissue engineering applications.

### 2.2 Proteomics Analysis

The decellularization process can significantly impact the retention of specific ECM components, potentially altering the materials’ biochemical composition.^[^ ^24^, ^25^, ^26^ ^]^ To further understand the effect of the demineralization and decellularization on the bone ECM composition, we conducted proteomic analysis comparing the protein composition of native porcine bone tissue and dECM, before pepsin digestion.

**Figure 2:**
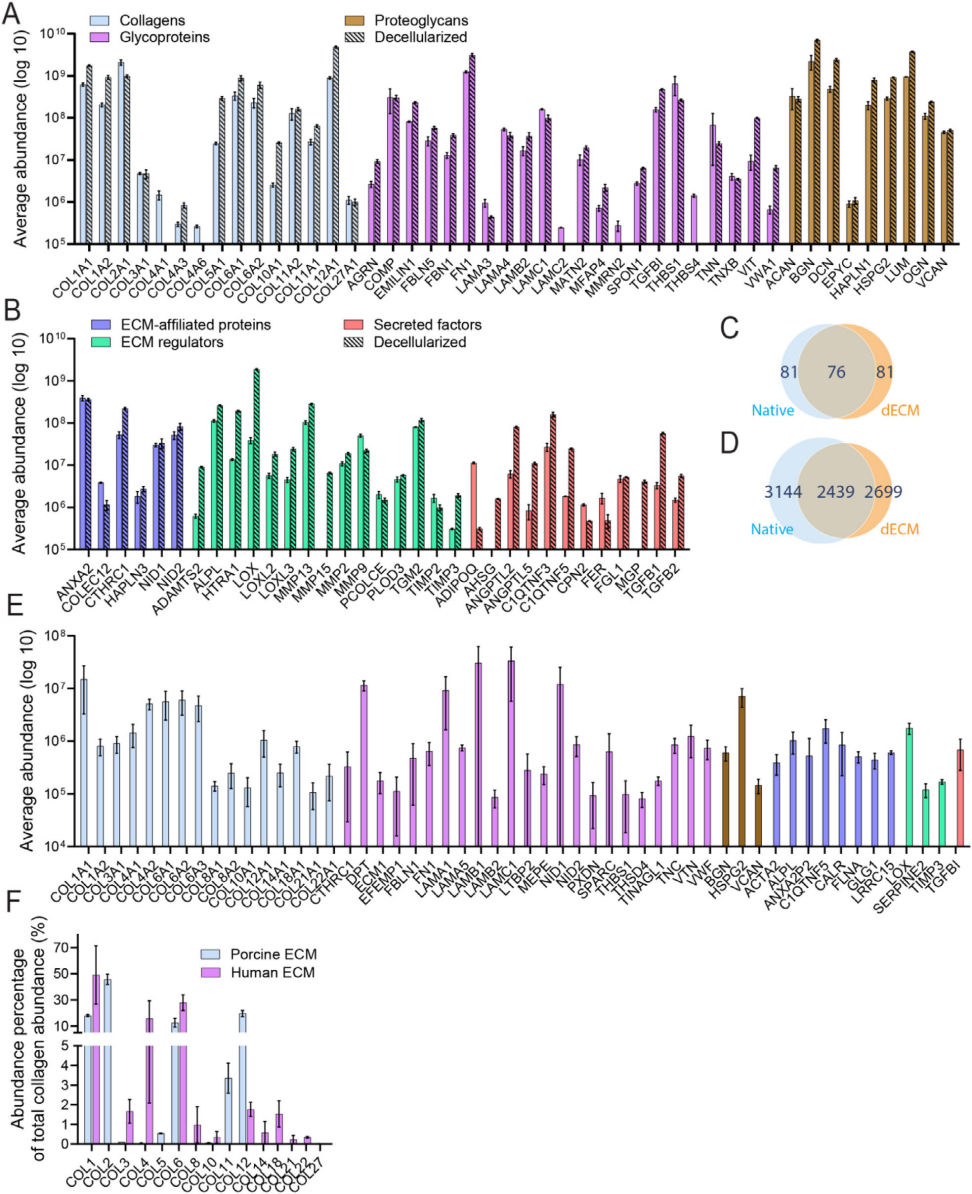
Mass spectrometry data of native and decellularized porcine bone tissue. A,B) The grouped abundance on log 10 scale of the matrisome proteins of the native porcine tissue (open) and the decellularized tissue (pattern) of A) the core matrisome proteins, including collagen, ECM Glycoproteins and proteoglycans, and B) the matrisome associated proteins, including ECM‐affiliated proteins, ECM regulator and secreted factors. The total number of C) matrisome proteins and D) proteins for the dECM and native porcine tissue. E) The grouped abundance on a log 10 scale of the matrisome proteins of native human tissue of the core and matrisome associated proteins. F) The percentage of abundance per collagen based on the total abundance of collagens of the native porcine and human tissue. All data shows the mean ± the standard deviation.

As expected, the most abundant proteins in both native and decellularized tissues were collagens, ECM glycoproteins, and proteoglycans (**Figure** **2**A,B; Table S1, Supporting Information). The native and decellularized tissues exhibited high expression of key extracellular matrix components, with biglycan (BGN), collagen type XII (COL12A1), lumican (LUM), and fibronectin (FN1) prominently present in both. In native tissue, collagen type I (COL1A1) and collagen type II (COL2A1) were also highly expressed, while in dECM, decorin (DCN) became more predominant, highlighting the preservation of essential ECM proteins despite decellularization (Figure 2A,B). We observed a higher abundance of matrisome proteins in dECM compared to native tissue, as indicated by an abundance ratio greater than 1 on a log2 scale (Table S1, Supporting Information), likely due to the enrichment of ECM components following decellularization.^[^ ^27^ ^]^ Additionally, certain proteins were detected only in the dECM. Since different decellularization methods enrich distinct ECM components, the specific protocol used can influence which proteins are retained and identified, and therefore contribute to the greater number of proteins observed in the dECM compared to the native tissue.^[^ ^27^, ^28^ ^]^ This enrichment of ECM proteins is likely due to the depletion of abundant cellular proteins present in native tissue, which often mask the detection of lower‐abundance matrix proteins.^[^ ^27^, ^28^ ^]^ Collagen type I, composed of COL1A1 and COL1A2, is the primary structural component of bone ECM, constituting 90% of the organic matrix alongside smaller amounts of types III and V.^[^ ^1^, ^29^ ^]^ The analysis confirmed the abundant presence of collagen type I in the native and dECM tissue. Notably, collagen type IV, a non‐fibrillar collagen that supports the basement membrane for osteogenic cells,^[^ ^30^ ^]^ has been removed during decellularization. We found 81 matrisome proteins in both native and decellularized bone tissue datasets, with 76 overlapping (Figure 2C). The dECM dataset contained 41 core‐matrisome proteins (collagens, glycoproteins, and proteoglycans) and 33 matrisome‐associated proteins (ECM regulators, secreted factors, and ECM‐affiliated proteins), while the native tissue included 46 core‐matrisome proteins and 29 matrisome‐associated proteins (Figure 2). The proteins were identified using the ECM‐specific MatrisomeDB 2.0 database.^[^ ^31^ ^]^ Collectively, these data suggest that the overall composition of matrisome proteins remains comparable between native and dECM bone tissue, suggesting that the ECM is largely preserved during demineralization and decellularization.

When comparing native porcine and human tissue, 25 shared ECM proteins were identified, reflecting a comparable proteomic profile of core matrisome and matrisome‐affiliated proteins (Figure 2A,B,E). 10 collagens were detected in porcine tissue and 11 in human tissue, with 6 overlapping, including the core structural collagens type I and III. Collagen type I was among the most abundant in both species (Figure 2F). The presence of collagen type II in porcine tissue suggests that the samples originated from young donors. In addition to collagens, other core matrisome glycoproteins, including laminin subunits and proteoglycans such as BGN, VCAN, and HSPG2, were detected in both porcine and human tissues (Figure 2F). Laminins are key components of the basement membrane and play a crucial role in regulating cell–matrix signaling, adhesion, and migration.^[^ ^32^ ^]^ Members of the TGF‐β superfamily, essential osteoinductive factors involved in bone development and osteogenic differentiation, were also present in both datasets.^[^ ^33^ ^]^ These shared ECM proteins underscore the proteomic similarity between porcine and human tissues, supporting the use of porcine‐derived ECM as a relevant and translationally applicable model for human bone tissue engineering.^[^ ^32^, ^33^ ^]^

### 2.3 Solubilization of Bone dECM and Synthesis of Methacrylated dECM (dECM‐MA)

After optimizing demineralization and decellularization conditions and characterizing the dECM matrisome, we aimed to establish the optimal enzymatic solubilization protocol and identify the minimal pepsin concentration required to achieve complete dECM solubilization within 92 h. SDS‐PAGE analysis and solubilized protein quantification in the dECM digests confirmed that a pepsin concentration of 0.5 mg mL^−1^ was sufficient to solubilize the dECM and produce a highly viscous and slightly opaque digest, while minimizing pepsin contamination compared to higher concentrations of 1 and 2 mg mL*^−^* ^1^ pepsin (Figure S4, Supporting Information).

**Figure 3:**
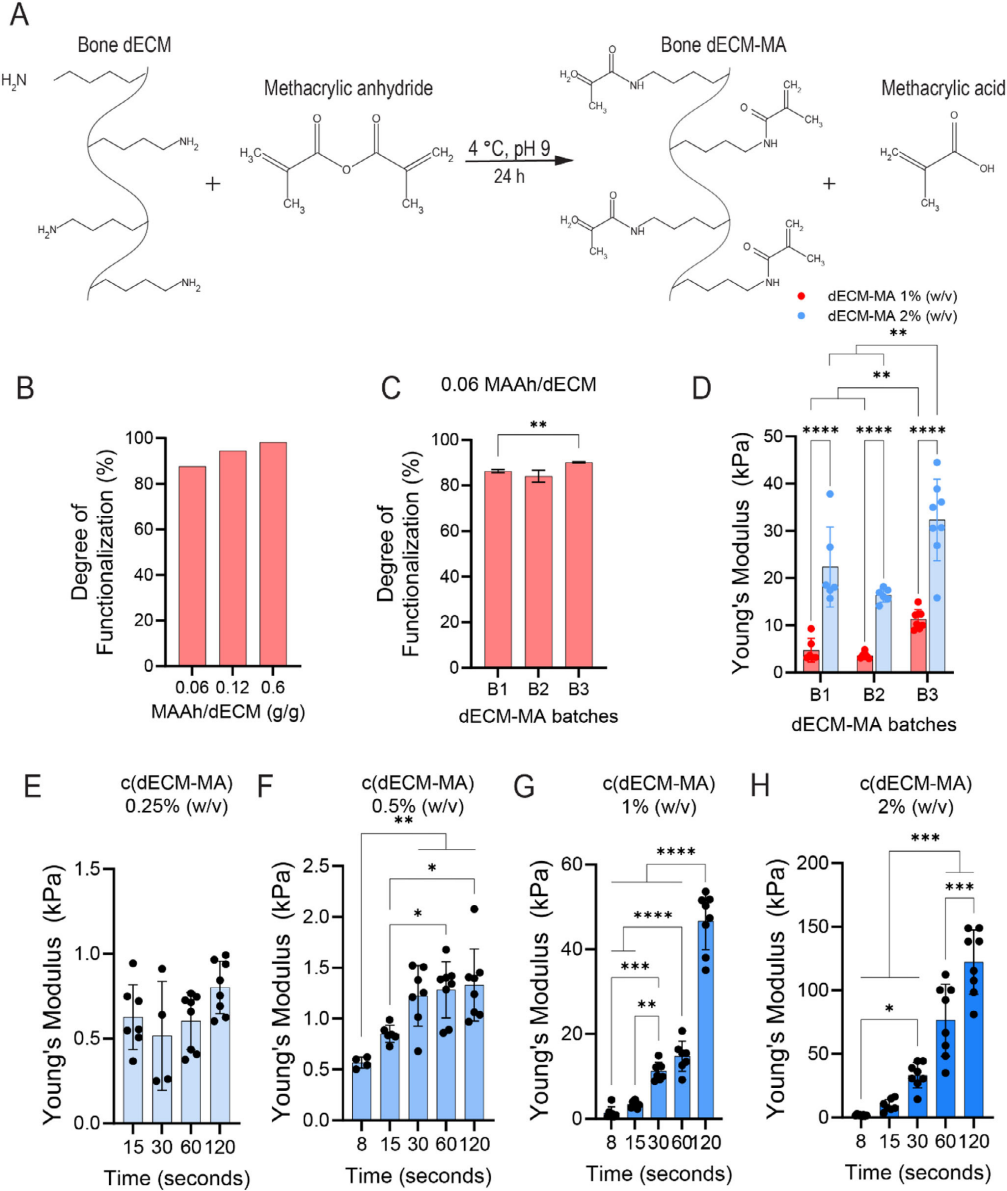
Mechanical properties of dECM‐MA hydrogels. A) The chemical reaction showing the functionalization of dECM with methacrylic anhydride at 4 °C, pH 9 to form dECM‐MA. B) Quantification of the degree of functionalization (DoF) of the free amines determined using a TNBS assay of the dECM decellularized with 3.4 m NaCl using a range of concentrations of 0.06, 0.12, and 0.6 g MAAh per g dECM. The data represent the DoF determined by calculating the difference in slope of free amines per concentration between dECM and dECM‐MA for 3 replicates (*n* = 3, technical replicates). C) The Do*F* for 3 dECM‐MA batches functionalized using 0.06 g MAAh per g dECM for 3 replicates (*n* = 3). Batches 1 and 2 (B1 and B2) were derived from the same decellularization process, whereas Batch 3 (B3) originated from a separate decellularization batch. D) Young's modulus was measured for the different batches in (C) at 1% and 2% (w/v) concentrations at 30 s of crosslinking. E–G) Young's modulus of dECM‐MA hydrogels with E) 0.25% (w/v), F) 0.5% (w/v), G) 1% (w/v), and H) 2% (w/v) dECM‐MA in PBS, measured across crosslinking times ranging from 8 to 120 s for at least 6 replicates (*n* = 6‐8). No hydrogel formation was observed within 8 s for 0.25% dECM‐MA. For C–H) Data represents mean ± SD. Statistical analysis was performed using one‐way ANOVA (C, E‐H) or two‐way ANOVA (d), with significance indicated by ^*^ *p* < 0.05, ^**^ *p* < 0.01, ^***^ *p* < 0.001, and ^****^ *p* < 0.0001.

We then chemically functionalized the solubilized dECM to introduce photocrosslinkable methacryloyl groups and synthesize dECM‐MA (**Figure** **3**A). Functionalization was performed using varying concentrations of methacrylic anhydride (MAAh), and the degree of functionalization (DoF) was quantified using a TNBS assay, which measures the availability of free amines primarily associated with lysine residues (Figure 3B,C). Functionalization with 0.06, 0.12, and 0.6 g MAAh per g dECM resulted in DoFs of 87%, 94%, and 98%, respectively (Figure 3B). Compared to GelMA, which typically exhibits DoFs between 20% and 85%, depending on the synthesis protocol,^[^ ^34^, ^35^ ^]^ the dECM‐MA in this study exhibited a substantially higher DoF. To preserve biologically relevant unfunctionalized lysine residues, which may play a critical role in ECM bioactivity, we selected 0.06 g MAAh per g dECM as the optimal concentration for downstream assays.

To evaluate batch‐to‐batch variability, 3 independently functionalized dECM‐MA batches were analyzed. One batch originated from a distinct decellularization process (B3), while the other two were derived from the same decellularization batch but functionalized separately (B1, B2) (Figure 3C). A small, significant difference was found in the DoF between batch 1 and batch 3.

The Young's compressive modulus was measured using an Instron MicroTester. Hydrogels were rapidly polymerized via radical crosslinking in the presence of 0.15% (w/v) LAP photoinitiator under 405 nm light, forming transparent, structurally stable hydrogels. A significant difference in Young's modulus was observed between the batch from the separate decellularization and the two batches from the shared decellularization, which was associated with differences in the DoFs (Figure 3C,D). Despite this variability, all batches consistently showed significant increases in stiffness with higher dECM‐MA concentrations, confirming the tunability of hydrogel mechanical properties through concentration adjustment. The batch‐to‐batch variation is minimized by pooling multiple donors per decellularization batch.

To further assess the tuneability of the mechanical properties, hydrogels were prepared at 0.25%, 0.5%, 1%, and 2% (w/v) dECM‐MA and crosslinked for 8, 15, 30, 60, or 120 s. The Young's modulus increased with both dECM‐MA concentration and crosslinking time (Figure 3A–D). Hydrogels with 0.5%, 1%, and 2% dECM‐MA exhibited a significant increase in stiffness over time (Figure 3B–D), while 0.25% dECM‐MA did not form hydrogels within 8 s (Figure 3A). By adjusting dECM‐MA concentration and crosslinking duration, hydrogel stiffness was precisely controlled over a broad range (≈0.5–120 kPa), enabling optimization for soft tissue and bone‐mimetic applications.

### 2.4 Cytocompatibility of dECM‐MA Hydrogels

The mechanical properties of biomaterials play a crucial role in regulating cell behavior and differentiation.^[^ ^36^, ^37^, ^38^ ^]^ To investigate the effect of matrix stiffness on osteoblast behavior, human pre‐osteoblasts (hOBs) were encapsulated in dECM‐MA hydrogels with Young's moduli of 5, 10, and 20 kPa, respectively. Previous studies have shown that lower‐stiffness GelMA hydrogels (6.3 kPa) promote mineralization more effectively than higher‐stiffness GelMA hydrogels (40.2 kPa).]^[^ ^37^ ^]^ Based on this, it was hypothesized that softer dECM‐MA hydrogels (5 kPa) would enhance mineralization compared to stiffer dECM‐MA formulations (10 and 20 kPa).

To test this hypothesis, hOBs were encapsulated in dECM‐MA hydrogels with Young's moduli of 5, 10, and 20 kPa, respectively, with 5 kPa GelMA hydrogels serving as a non‐tissue‐specific control.^[^ ^12^ ^]^ As demonstrated in the mechanical data (Figure 3), Young's modulus was tuned by adjusting both crosslinking time and dECM‐MA concentration. Hydrogels with a Young's modulus of 5, 10, and 20 kPa were obtained by using 1% dECM‐MA crosslinked for 17 s, 1% dECM‐MA crosslinked for 30 s, and 2% dECM‐MA crosslinked for 22 s, respectively.

**Figure 4:**
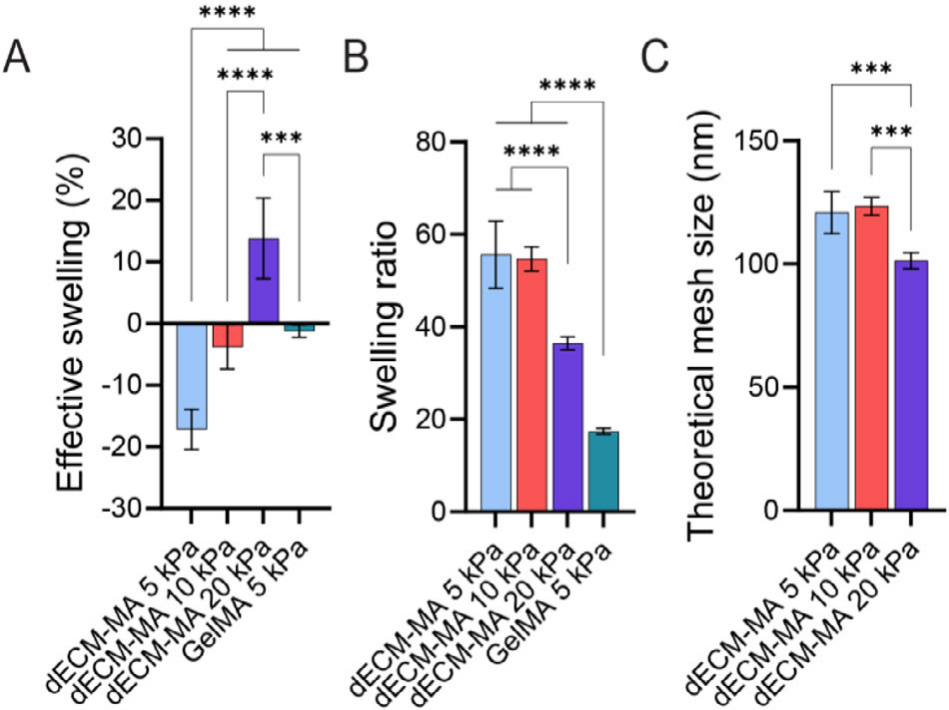
Swelling properties and theoretical mesh size of dECM‐MA hydrogels. A) The effective swelling of 5, 10, and 20 kPa dECM‐MA and 5 kPa GelMA. B) The swelling ratio of 5, 10, and 20 kPa dECM‐MA and 5 kPa GelMA. C) The theoretical mesh size of 5, 10, and 20 kPa dECM‐MA based on the swelling ratio (B) and characteristic material parameters of gelatin. Data represents mean ± SD. Statistical analysis was performed using one‐way ANOVA with significance indicated by ^***^ *p* < 0.001, and ^****^ *p* < 0.0001.

In addition to stiffness, other mechanical properties such as material porosity play an important role in osteogenic differentiation, influencing cell morphology and cell–substrate interactions.^[^ ^39^ ^]^ Therefore, the effective swelling and swelling ratio were determined to calculate the theoretical mesh size of the dECM‐MA hydrogels (**Figure** **4**). The effective swelling was negative for the 5 kPa hydrogels and increased with stiffness, with the 10 kPa dECM‐MA and GelMA hydrogels exhibiting comparable swelling (Figure 4A). A significantly smaller mesh size was observed for the 20 kPa dECM‐MA hydrogel compared to the 5 and 10 kPa hydrogels (Figure 4B), demonstrating the intrinsic coupling between hydrogel stiffness, mesh size, and polymer concentration, where increasing stiffness corresponds to a decrease in mesh size.

**Figure 5:**
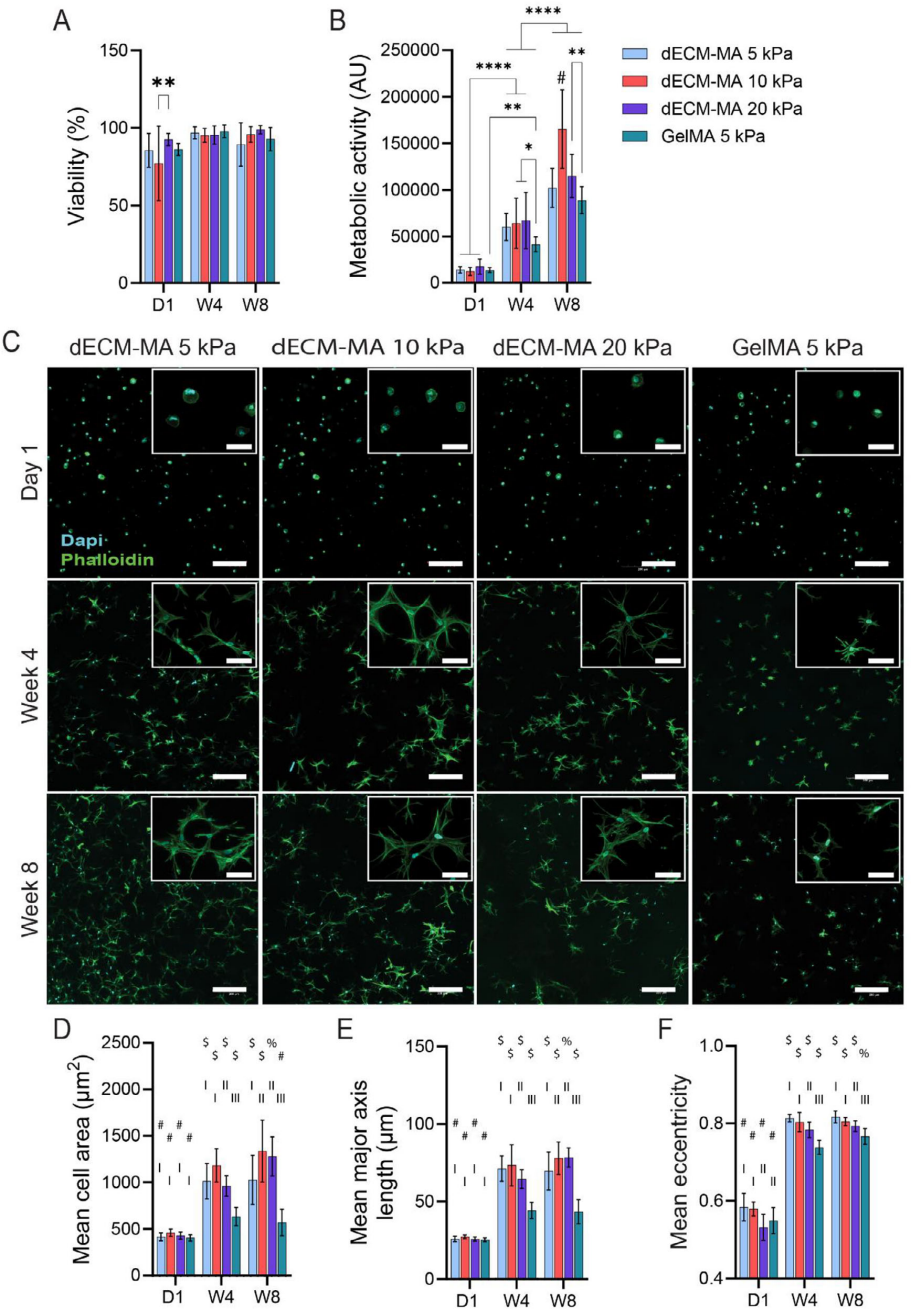
Cytocompatibility and morphology analysis of hOBs encapsulated in dECM‐MA and GelMA hydrogels. hOBs were seeded at 2 × 10^6^ cells per mL in 25 µl dECM‐MA or GelMA hydrogels. A) Quantified viability using CellProfiler software, analyzing 3 regions of interest of 3 hydrogels on day 1, week 4, and week 8. B) Metabolic activity was determined using PrestoBlue assay, showing fluorescence at 570 nm (arbitrary units). All data represent the mean ± the SD for 18 replicates. (*n* = 6, 2 biological replicates, 2 hydrogels per bio rep). Statistical analysis was performed using a two‐way ANOVA, with significance indicated by ^*^ *p* < 0.05, ^**^ *p* < 0.01, and ^****^ *p* < 0.0001. # indicates a significant difference (*p*<0.0001) to all other groups per timepoint. C) Morphology of hOBs in dECM‐MA and GelMA control stained with DAPI (nuclei in blue) and phalloidin (f‐actin stained in green) on day 1, weeks 4 and 8 using a 10x magnification. Scalebar = 200 µm. In the corner, there is a zoomed‐in display of the hOBs using a 30x magnification with a scalebar = 50 µm. To quantify the morphology, D) the mean cell area, E) the mean major axis length, and F) the mean cell eccentricity of all cells per image were determined using a CellProfiler pipeline, with eccentricity = 0 meaning a perfect circle. D–F) The data shows the mean ± SD for 12 replicates. (*n* = 4, 2 biological replicates, 2 hydrogels per bio rep, 3 ROI per hydrogel). Statistical analysis was performed using a two‐way ANOVA, with significant differences between timepoints are given with symbols, and the difference between groups per timepoint with Roman letters.

The viability of hOBs remained high across all dECM‐MA hydrogels, with average viabilities of 92%, 89%, and 95% for 5, 10, and 20 kPa hydrogels at week 8, respectively, comparable to the 5 kPa GelMA control (**Figure** **5**A; representative images Figure S5, Supporting Information). Metabolic activity progressively increased from week 4 to week 8, with hOBs in the 10 kPa dECM‐MA hydrogel showing significantly higher metabolic activity at week 8 compared to the other dECM‐MA formulations and the GelMA control (Figure 5B). These findings indicate that while matrix stiffness and composition did not affect long‐term cell viability, they influenced metabolic activity.

Fluorescent staining suggested that hOBs encapsulated in dECM‐MA hydrogels exhibited a larger, more spread morphology with extended lamellipodia and intercellular connections, in contrast to the rounded morphology observed in the GelMA control (Figure 5C). These morphological differences are indicative of osteoblast differentiation, as the transition from pre‐osteoblasts to osteoblasts and ultimately osteocytes is characterized by changes in cell area, process formation, and cytoskeletal organization.^[^ ^40^ ^]^ Comparable morphological adaptations have been reported in primary mouse osteoblasts cultured in collagen gels, where cells adopted a stellate morphology and upregulated osteocyte‐specific genes (*DMP1, SOST, FGF23*) relative to 2D cultures.^[^ ^41^ ^]^ The morphology of hOBs in dECM‐MA hydrogels closely resembled this osteocyte‐like phenotype (Figure 5C), suggesting that matrix stiffness and ECM composition play a role in guiding osteoblast differentiation.

Quantitative analysis using CellProfiler confirmed significant increases in mean cell area and major axis length over time in dECM‐MA hydrogels compared to day 1 (Figure 5D,E). By weeks 4 and 8, both parameters were significantly greater than in the GelMA control, indicating enhanced cell spreading and elongation. Additionally, cell eccentricity increased over time (Figure 5F), reflecting a shift from a circular to an elongated morphology, consistent with osteoblast‐to‐osteocyte differentiation.

**Figure 6:**
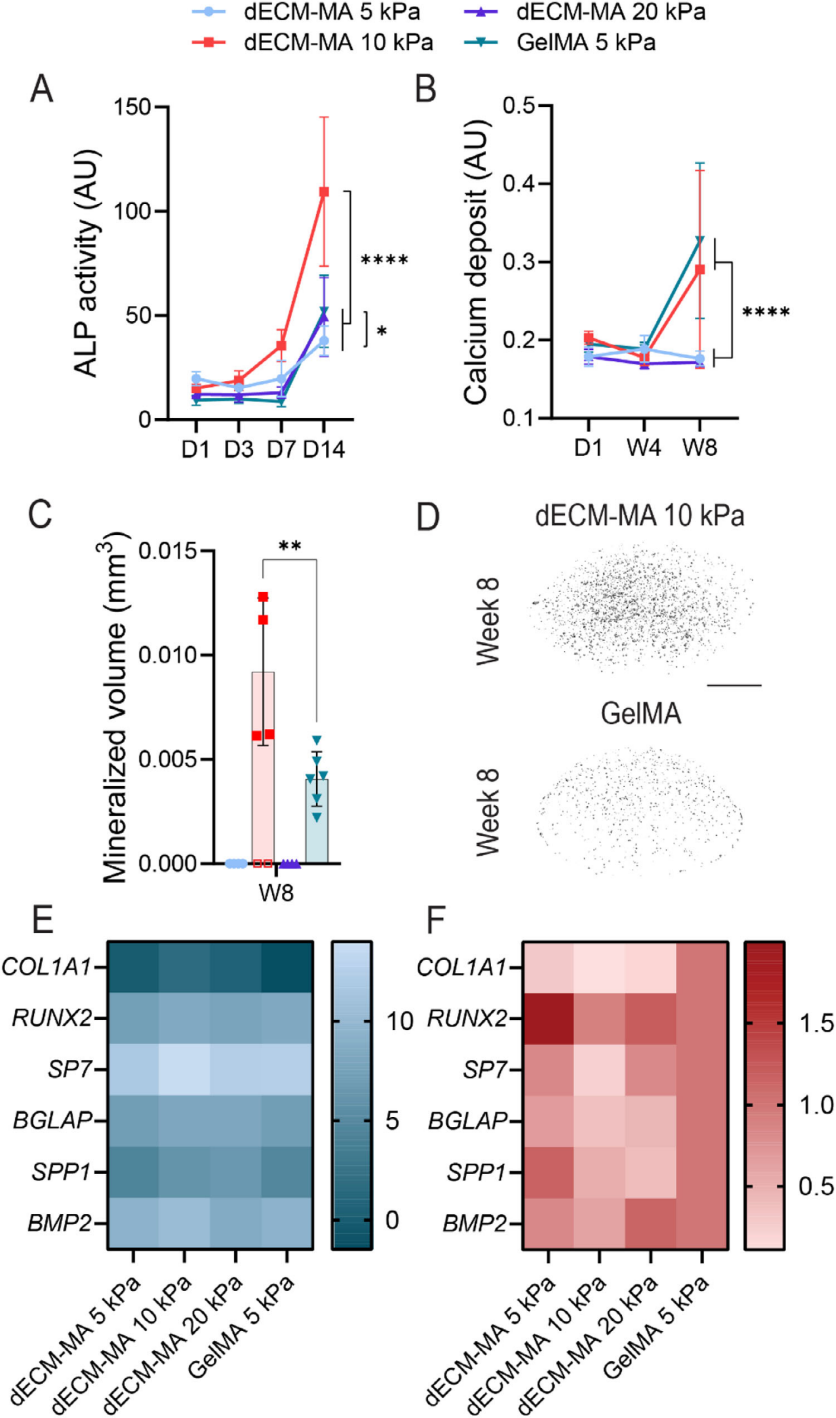
Osteogenic differentiation of pre‐HOBs in dECM and GelMA hydrogels. A) Alkaline phosphatase (ALP) activity, an early osteogenic marker, was measured at days 1, 3, 7, and 14. B) Calcium deposition was quantified using Alizarin Red S extraction, showing mineral accumulation over time. Data represent the mean ± SD for 18 replicates (*n* = 6, 2 biological replicates, 3 hydrogels per biological replicate). Statistical analysis was performed using a two‐way ANOVA, with significance indicated by ^*^< 0.05 and ^***^ *p* < 0.0001. C) Mineralized ECM volume in the hydrogels was quantified via µCT analysis at week 8. Data represents SD for 4–6 replicates (*n* = 4–6, 2 biological replicates, 2 or 3 hydrogels per biological replicate). Statistical analysis was performed using a one‐way ANOVA, with significance indicated by ^**^ *p* < 0.001, where the open square data points are left out of the statistical analysis. D) Representative µCT images of mineralization in dECM‐MA 10 kPa and GelMA hydrogels at week 8. Scale bar = 1 mm. The dECM‐MA 10 kPa group exhibited high variability, with the image shown representing a hydrogel with high mineralized volume (corresponding to panel c). E) Gene expression analysis shows ΔCT values of COL1A1, RUNX2, SP7, BGLAP (osteocalcin), SPP1 (osteopontin), and BMP2, normalized to RPLP32. F) Fold‐change gene expression was calculated using and adaptation of the ΔΔCT method (see methods), normalized to RPLP32, and the GelMA control, where no significant differences were found per gene between dECM‐MA and GelMA hydrogels using a one‐way ANOVA analysis. Primer sequences are listed in the methods section.

Next, osteoblast differentiation in dECM‐MA hydrogels and the GelMA control was assessed by measuring alkaline phosphatase (ALP) activity and calcium deposition. ALP is a key enzyme expressed by osteoblasts that initiates mineralization and serves as an early osteogenic differentiation marker.^[^ ^42^, ^43^ ^]^ ALP activity was measured on days 1, 3, 7, and 14, revealing significantly higher activity in the dECM‐MA 10 kPa hydrogels compared to the other groups (**Figure** **6**A). However, high variability among dECM‐MA 10 kPa replicates resulted in large standard deviations, indicating inconsistent ALP expression across samples.

To quantify calcium deposition, hydrogels were stained with Alizarin Red and analyzed via µCT imaging on day 1, week 4, and week 8. Semi‐quantitative analysis of Alizarin Red extraction showed significantly higher calcium deposition in dECM‐MA 10 kPa and GelMA compared to other groups (Figure 6B). This was consistent with µCT imaging, which revealed increased mineralized ECM volume in these conditions (Figure 6C,D). However, mineral deposition in dECM‐MA 10 kPa hydrogels was highly variable, with four of the six replicates showing mineralization. Analysis of the mineralized constructs revealed a significant increase in mineralization in the 10 kPa dECM‐MA hydrogels compared to GelMA (Figure 6C).

These findings suggest that dECM‐MA 10 kPa hydrogels promote osteoblast differentiation and mineralization, although the high variability in mineral deposition warrants further investigation to determine the underlying cause.

To further investigate differences in hOB morphology and differentiation between dECM‐MA and GelMA hydrogels, mRNA expression analysis and immunohistochemistry were performed to assess the expression of osteoblast and osteocyte markers at both the RNA and protein levels (Figure 6E,F). Osteoblast differentiation is a sequential process characterized by the upregulation of specific markers. *RUNX2* and *SP7* (Osterix) are early osteogenic transcription factors, with *SP7* acting downstream of *RUNX2* to regulate osteoblast maturation. *RUNX2* activates the expression of osteoblast‐associated genes, including Collagen Type I (*COL1A1*), Osteopontin (*SPP1*), and Osteocalcin (*BGLAP*).^[^ ^40^ ^]^ As differentiation progresses, osteoblasts secrete ECM proteins such as COL1A1 and BMPs, which support bone matrix formation.^[^ ^40^ ^]^ Late‐stage osteoblasts and osteocytes express *BGLAP*, *SPP1*, and *COL1A1*, while DMP1 serves as an early and mature osteocyte marker, respectively.^[^ ^40^, ^42^, ^44^ ^]^

Gene expression analysis, normalized to *RPLP32*, showed that *COL1A1* had the highest expression across all groups (Figure 6E). As *COL1A1* and *BGLAP* are key regulators of bone matrix formation, their upregulation indicates that both dECM‐MA and GelMA hydrogels support osteoblast differentiation.^[^ ^44^ ^]^ However, fold‐change analysis comparing dECM‐MA to GelMA revealed no significant differences in gene expression (Figure 6F). Increased expression of *RUNX2*, *BGLAP*, and *SPP1* in both groups confirms osteoblast differentiation (Figure 6E), suggesting that dECM‐MA and GelMA hydrogels support osteoblast differentiation.

**Figure 7:**
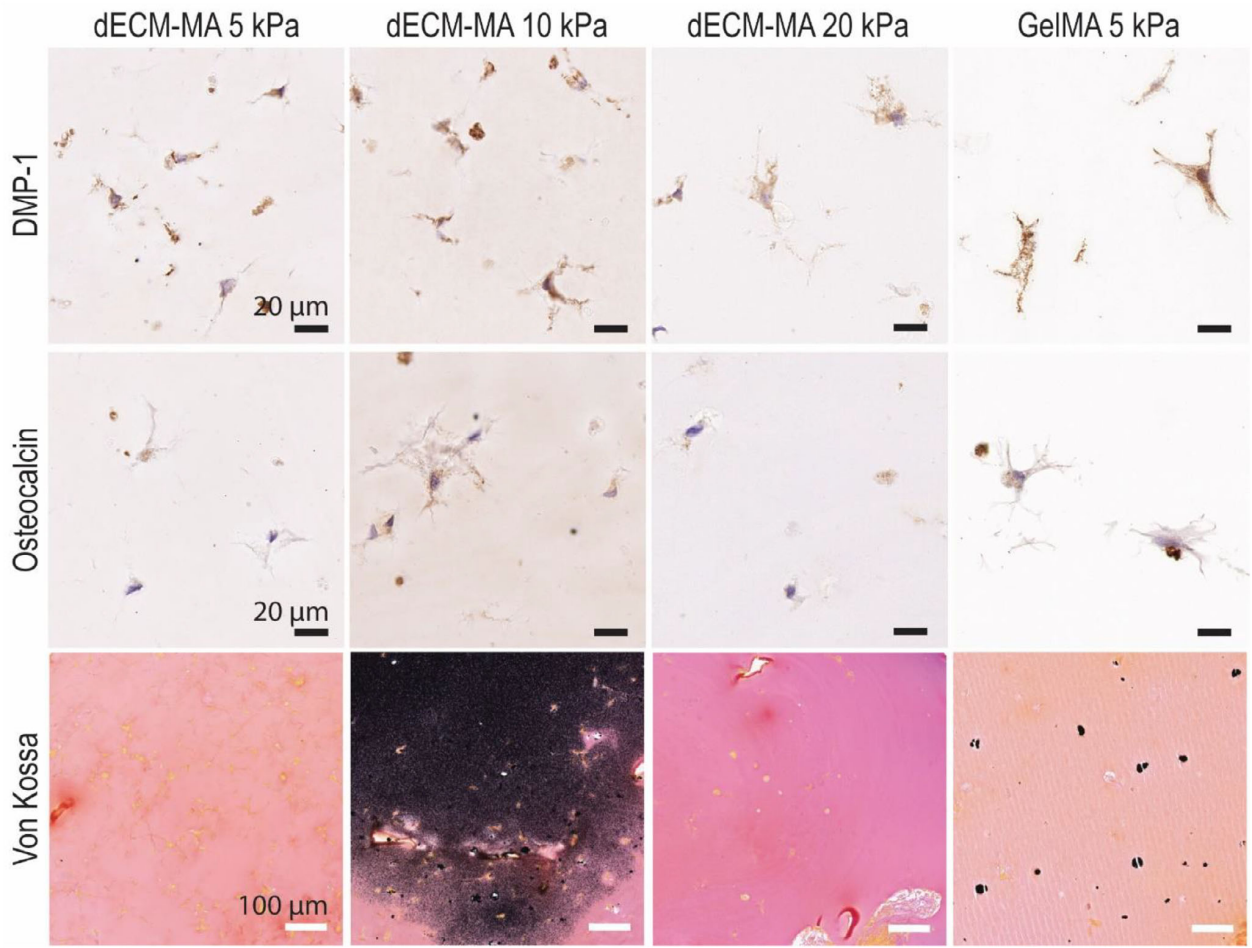
Immunohistochemical analysis of osteogenic differentiation in dECM‐MA and GelMA hydrogels. Representative immunohistochemistry images of DMP‐1, osteocalcin, and Von Kossa staining for mineralization, in hOB‐laden dECM‐MA (5, 10, and 20 kPa) and GelMA (5 kPa) hydrogels. Sections were 4 µm thick and imaged at 40× magnification. Black scale bars = 20 µm (DMP‐1, osteocalcin) and the white scale bar = 100 µm (Von Kossa).

DMP1 plays a critical role in phosphate regulation and is expressed by osteocytes.^[^ ^45^ ^]^ Given that cells with extended lamellipodia exhibit morphological features consistent with osteocyte differentiation, it was hypothesized that they would also show higher osteocyte marker expression.^[^ ^41^, ^43^ ^]^ Immunohistochemical analysis confirmed the expression of osteocalcin as well as DMP1 across all hydrogel formulations (**Figure** **7**). The presence of DMP1 suggests maturation of osteoblasts into osteocytes (Figure 7). When looking at the Collagen I staining, we found high background levels in acellular control hydrogels. However, the staining intensity in the 10 kPa dECM‐MA and GelMA hydrogels was significantly higher than the background signal (Figure S6b, Supporting Information).

To assess matrix mineralization, the hydrogels were stained with Von Kossa. Consistent with µCT analysis, dECM‐MA 10 kPa and GelMA hydrogels exhibited positive staining, indicating calcium phosphate deposition (Figure 7). However, high variability in Von Kossa staining was observed in dECM‐MA 10 kPa, with some biological replicates showing strong mineralization while others exhibited little to none (Figure S7, Supporting Information).

These findings demonstrate that both 10 kPa dECM‐MA and GelMA hydrogels support osteoblast differentiation, matrix deposition, and mineralization, though variability in mineralization outcomes suggests that additional factors may influence the extent of calcium phosphate deposition.

## 3 Discussion

Biomimetic hydrogels with adaptable physicochemical properties and biocompatibility can mimic the native ECM, supporting cellular adhesion, proliferation, and differentiation in tissue engineering applications. These hydrogels provide a supportive microenvironment for cellular growth and actively promote osteoblastic differentiation.^[^ ^2^ ^]^ Among these, photocrosslinkable dECM hydrogels closely replicate the native tissue environment while allowing precise modulation of mechanical properties.^[^ ^46^ ^]^ In this study, demineralization and decellularization processes were optimized to produce decellularized trabecular bone tissue. The resulting bone dECM was solubilized and chemically functionalized with methacryloyl groups to generate photocrosslinkable bone‐derived ECM hydrogels. These hydrogels were evaluated for their ECM composition, mechanical characteristics, and cytocompatibility, with a focus on their ability to support osteogenic differentiation.

We successfully demineralized and decellularized the bone ECM, with most DNA removed during EDTA‐facilitated demineralization (Figure 1). By binding Ca^2^⁺ and Mg^2^⁺, EDTA disrupts cell‐ECM adhesions, enhancing decellularization.^[^ ^8^ ^]^ Osmotic shock was then applied to further remove cellular components, ensuring minimal toxicity and effective clearance of residual chemicals.^[^ ^8^ ^]^ The DNA content of the dECM, ≈50 ng.mg^−1^, falls close to the commonly accepted threshold (<50 ng.mg^−1^ per tissue dry weight) for successful decellularization, indicating effective removal of cellular material.^[^ ^47^ ^]^ Decellularization methods can alter ECM composition, potentially impacting the bioactivity and mechanical properties of the resulting hydrogel.^[^ ^46^, ^48^ ^]^ Given the importance of preserving the ECM composition during these processes, histological and proteomic analyses were conducted to assess the preservation of major ECM components after decellularization.

Proteomic analysis showed that the matrisome of the native porcine trabecular bone tissue and the dECM retained a comparable protein composition, including key ECM components such as collagens, core matrisome proteins, and laminins (Figure 2A,B). Laminins are important ECM regulators that regulate cell behavior and matrix‐mediated signaling.^[^ ^32^ ^]^ Despite minor differences between the native and decellularized tissue, the aim was to mimic the complex composition of the ECM. This analysis confirmed the retention of major ECM components, crucial for ensuring the biological activity of the hydrogel and mimicking native bone tissue. One of the challenges of proteomics analysis of bone tissue is the solubilization of the tissue for analysis, due to the mineral and collagen content of the tissue.^[^ ^49^ ^]^ The PhyloBone database identified 449 bone ECM proteins in humans and 251 in pigs, without specifying whether they were derived from trabecular, cortical, or both types of bone, whereas we found 86 unique ECM proteins (Figure 2A,B).^[^ ^50^ ^]^ However, the number of matrisome proteins that were identified is comparable to a similar proteomics study analyzing the bone ECM.^[^ ^12^ ^]^

The proteomics analysis confirmed a high degree of similarity between the porcine and human protein profiles (Figure 2E,F). Porcine tissue is a widely used source for decellularized extracellular matrix (dECM) due to its availability, cost‐effectiveness, and anatomical similarity to human tissues.^[^ ^51^ ^]^ Unlike human donor tissues, which often originate from older individuals with variable health conditions, porcine tissues are typically harvested from young, healthy animals raised under standardized conditions, resulting in more consistent ECM quality.^[^ ^52^ ^]^ Numerous studies have demonstrated the osteogenic potential of porcine‐derived ECM in both in vitro and in vivo bone regeneration models.^[^ ^53^, ^54^, ^55^, ^56^ ^]^ Although human‐derived ECM can be preferred to minimize immunogenic risk,^[^ ^57^ ^]^ in *vivo* studies have shown that porcine ECM hydrogels elicit only a minimal immune response and effectively support bone formation.^[^ ^52^ ^]^ Comparative studies have shown that porcine and human bone ECMs share highly conserved structural proteins, such as type I collagen, with minor differences primarily attributed to donor age and health status.^[^ ^52^ ^]^ Supporting this, analyses of treated dentin matrices from human and porcine sources also revealed comparable proteomic profiles and regenerative potential, highlighting similar bioactivity across species.^[^ ^58^ ^]^ Together, these findings support the use of porcine‐derived ECM as a consistent, bioactive alternative to human ECM for bone tissue engineering applications.

Bone‐derived ECM hydrogels have been explored as potential clinical delivery systems.^[^ ^10^ ^]^ Early studies showed increased proliferation of mouse primary calvarial cells. Since then, porcine and human bone ECM‐derived hydrogels demonstrated enhanced mineralization compared to gelatin gels, as well as improved in vivo bone formation.^[^ ^12^, ^52^, ^53^ ^]^ However, the hydrogel gelation of these dECM hydrogels depends on temperature and pH. Functionalizing bone‐derived dECM enabled the development of rapidly crosslinked hydrogels with tunable mechanical properties. BoneMA, the first functionalized bone‐derived dECM hydrogel, improved tunability but had a limited Young's modulus range of 0.9–1.5 kPa.^[^ ^13^ ^]^ In contrast, dECM‐MA hydrogels exhibited a broader stiffness range of ≈0.5–120 kPa (Figure 3E–H), allowing for better biomechanical customization and optimization for potential disease modelling. Batch‐to‐batch variation of the dECM‐MA hydrogels was analyzed, and small significant differences in DoF and Young's modulus were found between batches (Figure 3C,D). Batch‐to‐batch variability can be reduced by pooling donors and standardizing processing procedures, while quality control measures, such as assessing the DoF and performing mechanical testing, are essential to ensure reproducibility across hydrogel preparations.

While biomimetic hydrogels are typically designed to match native tissue mechanics, studies suggest that lower stiffness enhances mineralization in osteoblast cultures, as seen in GelMA hydrogels (6.3–40.2 kPa).^[^^[^ ^37^ ^]^ Similarly, osteogenic differentiation of mesenchymal stem cells (MSCs) has been reported in environments of 11–30 kPa, whereas lower stiffness ranges promote adipocytic differentiation of the MSCs.^[^ ^59^ ^]^ Soft hydrogels can also support osteogenic differentiation when combined with biochemical cues, high‐affinity adhesive ligands, or degradability that facilitates cell spreading.^[^ ^60^, ^61^, ^62^ ^]^ Beyond stiffness, the mesh size or porosity of hydrogels also influences osteogenic differentiation. Increase in porosity has been associated with enhanced osteogenic differentiation^[^ ^63^ ^]^ as larger mesh sizes provide cells with the space needed to remodel the extracellular matrix, a process that helps regulate their differentiation.^[^^[^ ^37^, ^64^ ^]^ The mesh size, defined as the distance between adjacent crosslinks, is typically <100 nm in hydrogels, whereas bone tissue formation using scaffolds requires minimum pore sizes of ≈100 µm, ideally >300 µm.^[^ ^65^, ^66^, ^67^ ^]^ Therefore, macroporous hydrogels with pore sizes of 100–1000 µm have been developed and shown to promote osteogenesis.^[^ ^39^, ^64^ ^]^ Estimating the theoretical mesh size of dECM‐MA hydrogels provided insight into the mechanical and structural differences among 5, 10, and 20 kPa formulations (Figure 4). The superior osteogenic differentiation observed in the 10 kPa hydrogel compared to the 20 kPa condition may be in part attributed to increased mesh size. Consistent with this, we found that hOBs encapsulated in dECM‐MA hydrogels (10 kPa) and the GelMA control underwent osteogenic differentiation and mineralization, confirmed via µCT, Von Kossa staining, and Alizarin Red S staining (Figures 5 and 6).

Next, we examined the ability of dECM‐MA hydrogels to support osteogenic differentiation and mineralization in vitro. Bone tissue, primarily composed of minerals and type I collagen, features a highly mineralized ECM.^[^ ^12^ ^]^ To generate the dECM‐MA, the ECM was decellularized and demineralized; however, inducing a mineralized microenvironment is crucial for providing bioactive cues that promote osteogenic differentiation.^[^ ^68^ ^]^ While GelMA hydrogels mimic the ECM, exhibit high cytocompatibility, and support cell adhesion, proliferation, and differentiation, their inherent osteogenic capacity is limited.^[^ ^69^, ^70^ ^]^ To improve osteoconductivity, GelMA is often combined with osteoconductive materials, such as calcium phosphate ceramics or bioactive glasses.^[^ ^70^ ^]^ Similarly, decellularized ECM by itself or in combination with GelMA can serve as an osteoconductive matrix.^[^ ^7^, ^69^, ^71^ ^]^ Studies on collagen‐based and GelMA hydrogels have explored increasing osteoconductivity by supplementing osteogenic medium with soluble Ca^2^⁺ and PO₄^3^⁻, which enhances mineralization in soft hydrogels within four weeks.^[^ ^14^, ^37^, ^68^ ^]^ In contrast, human primary osteoprogenitors typically require 7–13 weeks for mineralization.^[^ ^72^ ^]^

In this study, we found that 10 kPa dECM‐MA and GelMA hydrogels supported hOB mineralization; however, the extent of mineralization was low compared to GelMA hydrogels with supplemented media found in the study described above (Figure 6D).^[^ ^1^, ^7^ ^]^

We hypothesize that the hOBs encapsulated and grown in hydrogels potentially have a greater requirement for osteogenic stimulation, above what was provided to them and what is commonly accepted in the literature as sufficient for predominantly 2D studies. The variability in mineralization outcomes likely reflects this insufficient stimulation rather than material inconsistencies, as no mechanical variability was observed within the 10 kPa hydrogel group. However, this cannot be definitively confirmed without individual stiffness measurements. Moreover, pooled hOBs were encapsulated in the hydrogels. While differentiation was initiated, as shown by ALP and gene expression data, mineralization was likely not fully supported under the current conditions. Future studies should explore media supplementation with soluble Ca^2^⁺ and PO₄^3^⁻ to enhance mineral deposition and reduce variability.^[^ ^14^, ^68^ ^]^

Cell–hydrogel interactions are influenced by several physicochemical properties, including stiffness, pore size, and surface chemistry, which in turn affect key cell functions such as spreading, proliferation, migration, and differentiation.^[^ ^73^ ^]^ Among the tested stiffnesses, 10 kPa dECM‐MA hydrogels supported the highest level of osteogenic differentiation. While no significant difference in mesh size was observed between 5 and 10 kPa hydrogels, both had significantly larger mesh sizes than 20 kPa hydrogels (Figure 4). Interestingly, hOBs in all dECM‐MA conditions showed greater cell spreading than those in GelMA controls (Figure 5C–F), likely due to the preserved bioactive cues in the dECM matrix.^[^ ^74^ ^]^ However, increased spreading did not consistently correlate with enhanced osteogenic differentiation across all dECM‐MA groups (Figure 6), suggesting that while cell–matrix interactions were improved, additional osteogenic cues are necessary to drive full maturation, as also reported in previous studies.^[^ ^60^, ^61^, ^62^, ^68^ ^]^ Proteomic analysis confirmed the retention of many ECM proteins and TGF‐ β units, yet key osteoinductive factors such as BMP‐2 were not quantified, and their bioactivity remains uncertain. This limits the interpretation of whether the observed osteogenic effects were primarily driven by mechanical cues, biochemical signals, or their interplay. These findings suggest that although 10 kPa dECM‐MA hydrogels support osteogenic commitment, differentiation and mineralization varied across stiffnesses, with lower activity at both 5 and 20 kPa. While mesh size may partly explain these differences, stiffness‐dependent effects likely involve more complex biophysical mechanisms. Further investigation is needed to clarify whether differentiation is driven predominantly by mechanical coupling or other biophysical and biochemical factors.

Beyond regenerative medicine, these functionalized bone‐derived dECM hydrogels have considerable potential for use in disease modeling and drug screening.^[^ ^75^ ^]^ Their tunable properties enable the creation of in vitro 3D models that can be optimized to model healthy and diseased tissues, including bone cancer models.^[^ ^46^, ^76^ ^]^ Biomimetic ECM scaffolds have been instrumental in advancing in vitro tumor models, providing insights into cancer metastasis by enabling the study of cancer cell interactions within a physiologically relevant matrix microenvironment.^[^ ^75^, ^77^ ^]^ Given that bone is a common site of metastasis for cancers such as breast and prostate cancer,^[^ ^78^ ^]^ as well as the primary site of osteosarcoma,^[^ ^79^ ^]^ bone‐derived dECM hydrogels offer a platform for studying both primary and metastatic bone cancers. The ability to fine‐tune mechanical properties further enhances their utility in modeling disease progression and screening potential therapeutic agents in a physiologically relevant setting.

## 4 Conclusion

In conclusion, we developed a photocrosslinkable, decellularized bone ECM hydrogel that retains key ECM components. By functionalizing the hydrogel, its mechanical properties can be tuned through concentration and crosslinking time, optimizing it for specific cell culture applications. The hydrogel demonstrated cytocompatibility, supporting hOB encapsulation and differentiation across varying stiffness conditions, with mineralization observed specifically in the 10 kPa dECM‐MA hydrogel. This system holds promise for future studies optimizing bone differentiation and potential disease modelling.

## 5 Experimental Section

### Demineralization and Decellularization of Trabecular Bone Matrix

Porcine femur bones were purchased from a local butcher, and their use was approved under QUT ethics approval AE TU 6842. To isolate trabecular bone, the femur bone ends were halved using a band saw, separating the trabecular matrix from the cortical bone. The trabecular bone was then cut into fragments using a bone cutter. The bone fragments were snap frozen using liquid nitrogen and ground into a fine powder using a coffee grinder (LCG350SIL, Breville).

The bone powder was demineralized at 4 °C using 10% ethylenediaminetetraacetic acid (EDTA, ThermoFisher) at pH 7.4 under continuous stirring for 7 days, with daily solution changes. The demineralization progress was monitored daily using micro‐computed tomography (µCT 50; Scanco Medical, Bruttisellen, Switzerland) at a 70 kV, 114 µA, 8W, a FOV/diameter of 10.2 mm, and a voxel size of 3 µm, following previously published methods.^[^ ^80^ ^]^ Based on the µCT analysis of bone fragments, complete demineralization of the bone powder was determined to occur within 3 days. The demineralized bone tissue was stored at −20 °C until further processing.

Lipid removal was performed using a 1:1 (v/v) solution of chloroform (Chem‐Supply, Australia) and methanol (ThermoFisher) for 2 h under constant agitation. The bone tissue was then washed three times with methanol and three times with ultrapure water to ensure complete solvent removal.

Decellularization was achieved through osmotic shock using 3.4 M sodium chloride (NaCl, Chem Supply, Australia). The bone tissue was placed in the NaCl solution at 4 °C for 4 days under continuous stirring, with daily solution changes. After decellularization, residual NaCl was removed by washing the samples six times with ultrapure water under agitation for 5–10 min per wash. Successful decellularization was confirmed by DNA quantification using a PicoGreen assay and histological evaluation with Hematoxylin and Eosin staining.

### DNA Quantification

The DNA content of native porcine trabecular bone tissue, demineralized trabecular bone tissue, and decellularized tissue at days 0, 2, 4, and 6 was quantified using the Quant‐iT PicoGreen dsDNA Assay Kit (ThermoFisher Scientific, USA) following the manufacturer's protocol. Bone tissue fragments were solubilized by incubation in 0.5 mg mL^−1^ proteinase K (Merck, Germany) prepared in phosphate‐buffered EDTA (PBE) buffer (pH 7.1) at 56 °C for 16–18 h. After digestion, samples were vortexed to ensure complete dissolution of the tissue. Fluorescence intensity was measured using the CLARIOstar microplate reader (BMG Labtech, Germany) at an excitation wavelength of 480 nm and an emission wavelength of 520 nm. DNA concentration in each sample was determined by comparison to a standard curve generated using lambda DNA provided with the assay kit.

### Glycosaminoglycan Quantification

The glycosaminoglycan (GAG) content of native trabecular bone tissue, demineralized trabecular bone tissue, and decellularized tissue at days 0, 2, 4, and 6 was quantified using the 1,9‐dimethyl methylene blue (DMMB) assay, following previously described protocols.^[^ ^3^ ^]^ Bone tissue fragments were solubilized in 0.5 mg mL^−1^ proteinase K (Merck, Germany) prepared in PBE buffer (pH 7.1) at 56 °C overnight. Samples were vortexed to ensure complete dissolution of the tissue before analysis. A dilution series of chondroitin sulfate (Sigma–Aldrich) standards (ranging from 100 to 3.125 µg mL^−1^) was prepared in PBE buffer. A standard curve was generated by measuring the ratio of absorbance at 525 and 595 nm using a second‐order polynomial curve fitting method. The absorbance at 525 and 595 nm was immediately measured using the CLARIOstar plate reader (BMG Labtech, Germany). The absorbance ratio (525 nm / 595 nm) was calculated for all samples and standards. The GAG concentration of each sample was interpolated from the standard curve using the following quadratic equation:

(1) $$\mathrm{GAG}\;\mathrm{concentration}=\frac{-b\pm \sqrt{b^{2}-4ac}}{2a}\;$$

where a, b, and c represent the shape, position, and roots of the second order polynomial function.

### Histology and Immunohistochemistry Decellularized Bone Tissue

Demineralized and decellularized bone fragments were fixed in 4% paraformaldehyde (PFA) overnight, embedded in 1% agarose gel, and processed into paraffin wax at 60 °C using the Leica ASP300S tissue processor (Leica, Germany) for 5 h. Embedding was performed with a Leica HistoCore Arcadia H + C paraffin embedding center (Leica, Germany). For histological analysis, 5 µm sections were prepared using a Leica RM2235 rotary microtome (Leica Biosystems, Germany). Sections were stained with Haematoxylin and Eosin (H&E), Safranin O, and Masson's Trichrome using standard protocols on a Leica Autostainer XL (Leica Biosystems).

Two stained slides, each containing two tissue sections with multiple bone fragments, were scanned at 40x magnification with a 3DHistech Scan II Brightfield slide scanner (3DHistech, Budapest, Hungary). Nuclei per tissue fragment were quantified using CellProfiler software and a pipeline adapted from Kim et al., 2020.^[^ ^4^ ^]^ A minimum of five regions of interest were analyzed per sample.

### Proteomics

Native porcine and human trabecular bone tissues were cut into smaller fragments using a bone cutter. Decellularized ECM (dECM) bone tissue was prepared as described above. For porcine samples, multiple donor bones were pooled and ground, while the human sample was derived from a single donor with three technical replicates. All samples were stored at a minimum of −20 °C prior to processing. Samples were lysed in a buffer containing 1% sodium deoxycholate, 100 mM Tris (pH 8.5), 10 mM tris(2‐carboxyethyl) phosphine (TCEP), and 40 mM 2‐chloroacetamide (CAA). Homogenization was performed at room temperature for 3 min using a bead mill with a single stainless‐steel ball bearing. Total protein concentration was measured using a Direct Detect infrared spectrometer (Merck). A total of 50 µg of protein was denatured by heating at 95 °C for 5 min. Protein digestion was performed using the Filter‐Aided Sample Preparation (FAST) protocol. Samples were diluted in Tris‐buffered urea and loaded onto 30 kDa Centricon filters. Proteins were digested overnight at 37 °C with 1 µg of trypsin in 75 µL of 50 mM ammonium bicarbonate.

The resulting peptide solutions were acidified to a final concentration of 0.5% (v/v) trifluoroacetic acid (TFA) and cleaned using C18 tips. Peptides were eluted in 80% (v/v) acetonitrile, vacuum dried, and resuspended in 0.5% (v/v) TFA prior to LC‐MS/MS analysis. A maximum of 1 µg of peptide was loaded for LC‐MS/MS analysis. Peptide separation was performed on an UltiMate 3000 RSLCnano system (Thermo Scientific) using a 50 cm Easy‐Spray C18 analytical column (Thermo Fisher, catalog numbers 160454 and ES803A). Mobile phases consisted of buffer A (0.5% TFA) and buffer B (80% acetonitrile). Peptides (1 µg per run) were initially loaded in 3% buffer B and separated using a linear gradient of 3–30% buffer B over 73 min, followed by a ramp from 30 to 95% over 17 min at a flow rate of 250 nL.min^−1^. Peptides were analyzed on a Q Exactive HF Orbitrap mass spectrometer (Thermo Scientific, USA) operating in positive ionization mode and optimized for high‐complexity peptide mixtures. Full MS scans from 350 to 1400 m/z were acquired at 60k resolution, with an AGC target of 3E6 and a maximum injection time of 100 ms. MS2 fragmentation was performed on the top 12 precursors, excluding 1+ and >7+ charged precursors. Precursor isolation width was 1.4 m/z, and normalized collision energy (NCE) was set to 27. MS2 scans ranged from 200 to 2000 m/z at 30k resolution, with an AGC target of 5E5, intensity threshold of 1E5, and a maximum injection time of 50 ms. The dynamic exclusion window was set to 30 s.

Raw files were analyzed in Proteome Discoverer (version 3.0), searching against the respective UniProt human or porcine proteomes, combined with the bovine proteome and an in‐house assembled protein contaminant database. Matrisome proteins were identified using the Matrisome Project database (https://hynes‐lab.mit.edu/resources/matrisome/fasta_files), applying both bovine and porcine FASTA files for the porcine bone samples. Identified proteins were further categorized using the MatrisomeDB 2.0 dataset.^[^ ^31^ ^]^ Search parameters included a precursor mass tolerance of 10 ppm and a fragment mass tolerance of 0.02 Da. A maximum of two missed trypsin cleavages was allowed. Carbamidomethylation of cysteine was set as a fixed modification, and oxidation of methionine as a variable modification. Searches were conducted using Chimerys. False discovery rate (FDR) analysis was performed using Percolator with a reversed concatenated database, and peptides were ranked by q‐value. FDR thresholds were 0.01 (strict) and 0.05 (relaxed). Sample input normalization was based on total peptide amount, and peptide abundance was determined by precursor intensity. Protein abundance was calculated from the summed intensity of all matching peptides. Protein fold changes were quantified based on pairwise peptide ratios. Imputation was performed using replicate‐based resampling, and group comparisons were conducted using a background‐based *t*‐test.

### Solubilization of the dECM

1% (w/v) decellularized bone ECM (dECM) was solubilized in 0.5 mg mL^−1^ pepsin (P6997, Sigma–Aldrich) dissolved in 0.01 m HCl. The mixture was continuously stirred at room temperature for 96 h. Protein content was measured by absorbance at 280 nm using a Nanodrop OneC spectrophotometer (ThermoFisher Scientific, USA). Pepsin was deactivated by adjusting the pH above 8 with 1 m NaOH, followed by lowering it to 2 with 1 m HCl at 4 °C. The solubilized ECM (sECM) was stored at pH 2 and 4 °C until further use.

### SDS Page

SDS‐PAGE was conducted following the Bio‐Rad protocol (Bulletin 6202) using 10% Mini‐PROTEAN TGX Stain‐Free Precast Gels (Bio‐Rad). Solubilized ECM (sECM) samples (0.5, 1, and 2 mg mL^−1^ pepsin) were diluted to 1 mg ECM per well and mixed 1:1 with sample buffer (4X Laemmli buffer with β‐mercaptoethanol, 9:1). The mixture was heated at 95 °C for 5 min.

A volume of 12.5 µL of each sample and 0.5 µL of protein ladder were loaded onto the gel. Electrophoresis was run at 4 °C, starting at 80 V for 15 min, followed by 150 V for ≈50 min, until the samples neared the gel's edge, using 1X running buffer (Bio‐Rad). The gel was stained with Silver Quick Stain, following the manufacturer's protocol (ThermoFisher Scientific, USA).

### Synthesis of Bone dECM‐MA

The sECM was functionalized at 4 °C under constant stirring while protected from light. The pH was adjusted to 9 using 1 m NaOH and 1 m HCl. To modulate the DoF, methacrylic anhydride (MAAh, Sigma–Aldrich) was added to the sECM at 0.06, 0.12, or 0.6 g MAAh per g ECM, maintaining the pH at 9 throughout the process. The reaction was stirred overnight to ensure complete functionalization.

To remove unreacted MAAh and sterilize the functionalized dECM (dECM‐MA), the solution was dialyzed using SnakeSkin Dialysis tubing (3.5K MWCO, ThermoFisher). The process included dialysis against 0.5% (v/v) chloroform in ultrapure water (pH 4) for 2 h to sterilize the dECM‐MA. This was followed by dialysis in ultrapure water at 4 °C under constant stirring, with daily water changes until the conductivity was stabilized. After dialysis, the dECM‐MA was frozen at −80 °C and subsequently freeze‐dried for storage.

### Degree of Functionalization

The DoF was determined with a TNBS assay following previously published protocols.^[^ ^81^ ^]^ The number of free amines of non‐functionalized dECM was compared to functionalized dECM‐MA. In short, a dilution series (1 in 2) of the dECM‐MA and the dECM solution in 0.1 m NaHCO3 buffer was prepared, ranging from 500 to 6.25 µg mL^−1^. 2,4,6‐Trinitrobenzenesulfonic acid (TNBS, stock 5% (w/v) solution, Sigma–Aldrich) was diluted in ultrapure water to prepare a 0.01% (w/v) TNBS solution. This solution was added to the well plate and mixed by placing the plate on a shaker for 5 min. The plate was incubated at 37 °C for 2 h protected from light. The absorbance was read at 335 nm using the CLARIOstar spectrophotometer (BMG Labtech, Germany) and plotted against the concentration. The DoF was calculated with the following formula:

(2) $$DoF=\left(1-\frac{m_{dECM-MA}}{m_{dECM}}\right)\;*100\%$$

Where m_dECM‐MA_ and m_dECM_ represent the slopes of the linear regression line of the plotted absorbance for the functionalized and non‐functionalized samples, respectively.

### Hydrogel Preparation

Prior to photo crosslinking, dECM‐MA solutions were diluted to 0.5% (w/v), 1% (w/v), and 2% (w/v) with PBS and 0.15% (w/v) lithium phenyl‐2,4,6‐trimethylbenzoylphosphinate photo initiator (LAP, Sigma–Aldrich). GelMA hydrogels (Porcine type A, Gelomics, Australia) were diluted to 5% (w/v) with PBS and 0.15% (w/v) LAP photo initiator. The resulting precursor solutions were crosslinked in a LunaCrosslinker photocrosslinking device (Gelomics, Australia) at 405 nm for different crosslinking times (15, 30 s, 1, 2, 4, and 8 min). To determine the Young's modulus, the hydrogels were crosslinked between a Teflon mold and a glass plate treated with Sigmacote (Sigma–Aldrich, Australia) to create hydrogels in the shape of a disk with a height of 1 mm. Hydrogels encapsulated with cells were directly crosslinked in non‐treated well plates.

### Mechanical Properties

The compressive moduli, or hydrogel stiffness, of the hydrogels were determined using an Instron MicroTester 5848 (Instron, Australia) equipped with a 5 N load cell following previously published protocols.^[^ ^82^ ^]^ Prior to testing, dECM‐MA gels were prepared 24 h in advance and allowed to swell in PBS overnight at 37°C with 5% CO_2_ in a humidified cell culture incubator. The hydrogels were imaged using a Stereoscope microscope (Nikon). Subsequently, the gels were placed in a hydrogel immersion bath containing PBS at 37 °C and compressed at a rate of 0.015 mm sec^−1^. The compressive modulus was determined as the slope of the linear region (0–15% strain) of the strain–stress curve.

### Swelling Properties and Theoretical Mesh Size

The swelling properties of the hydrogels were determined by measuring the post‐crosslinking weight (m_crosslinked_), the weight after overnight swelling (m_swollen_), and after freezing and lyophilizing the hydrogels (m_dry_) based on previously established protocols.^[^ ^76^, ^83^, ^84^ ^]^ The swelling properties were then calculated using the following formulae^[^ ^76^ ^]^:

(3) $$Effective\;swelling=\frac{\left(m_{swollen}-m_{crosslinked}\right)}{m_{crosslinked}}*100\%$$

(4) $$Swelling\;ratio\;\left(Q\right)=\frac{\left(m_{swollen}-m_{dry}\right)}{m_{dry}}$$

The theoretical mesh size (ξ) of the dECM‐MA hydrogels was estimated using the Flory–Rehner theory, based on the swelling ratio and characteristic parameters reported in previous protocols for comparable hydrogels.^[^ ^84^ ^]^ The values used for the calculations are summarized in **Table** **1**.

**Table 1:** Parameters to calculate the theoretical mesh size based on published values.^[^ ^84^ ^]^

Constant	Abbreviation	Value
Molecular weight of the repeating unit	M_r_	91.19 g.mol^−1^
Amino acid bond length	*l*	4.25 Å
Flory's characteristic ratio	C_n_	8.8785
Polymer density	ρ_p_	1.35 g.cm^−3^
PBS solvent density	ρ_s_	1.014 g.cm^−3^
Specific volume	𝑣̄	0.7407 mL g^−1^
Molar volume of solvent	V_1_	18.01 mL mol^−1^
Flory's Chi parameter	X_1_	0.497
Average molecular weight before crosslinks	M_n_	63,565.35 g.mol^−1^

The theoretical mesh size was calculated using formula (5) as previously published.^[^ ^84^ ^]^ The mesh size was calculated using the molecular weight of the repeating unit (M_r_), the amino acid bond length (*l*), the Flory's characteristic ratio (C_n_) and the molecular weight between crosslinks (M_c_) (**Table** **2**).

(5) $$Theoretical\;mesh\;size\;\;\left(\xi \right)={v_{2s}}^{-\frac{1}{3}}*l\;{\left(2\;\frac{M_{c}}{M_{r}}\;C_{n}\right)}^{\frac{1}{2}}$$

Where the molecular weight between crosslinks in g.mol^−1^ (M_c_) was calculated using Equations (6)‐(9). The relaxed and volumetric swelling were calculated using Equations (6) and (7) using the polymer density (ρ_p_) and the PBS solvent density (ρ_s_) (Table 2). The relaxed swelling ratio (*Q*_*m*(*r*)_) and equilibrium swelling ratio (*Q_m_*) were calculated using formula (4) using the wet weight after crosslinking (m_crosslinked_) instead of (m_swollen_) for the relaxed swelling ratio.

(6) $$Relaxed\;volumetric\;swelling\;\left(Q_{v\left(r\right)}\right)=1+\frac{\rho_{p}}{\rho_{s}}\left(Q_{m\left(r\right)}-1\right)$$

(7) $$Equilibrium\;volumetric\;swelling\;\left(Q_{v}\right)=1+\frac{\rho_{p}}{\rho_{s}}\left(Q_{m}-1\right)$$

**Table 2:** Primer sequences for PCR.

Gene	Forward	Backward
RPL32	CAGGGTTCGTAGAAGATTCAAGGG	CTTGGAGGAAACATTGTGAGCGATC
*Col1A1*	GTACAGAACGGCCTCAGGTACCATGA	GGGCAGTTCTTGGTCTCGTCACAGAT
*RUNX2*	CCCAGTATGAGAGTAGGTGTCC	GGGTAAGACTGGTCATAGGACC
*SP7*	CGGCAAGAGGTTCACTCGTTCG	TGGAGCAGAGCAGGCAGGTG
*BGLAP*	AGCGAGGAGTTGAATGGTGCATAC	AATCTGGACTGCTTGTGGCTGTG
*SPP1*	GTGCAGAGTCCAGCAAAGGT	TCAGCCAACTCGTCACAGTC
*BMP2*	CCCACTTGGAGGAGAAACAA	GCTGTTTGTGTTTGGCTTGA

The relaxed polymer volume fraction (*v*_2*r*_) and equilibrium polymer volume fraction (*v*_2*s*_) were calculated using formula (8) and the relaxed and equilibrium swelling ratio (*Q*_*m*(*r*)_ and *Q_m_*).

(8) $$Polymer\;volume\;fraction\;\left(v\right)=\frac{1}{Q}\;$$

The molecular weight between crosslinks (M_c_) was calculated using the Flory‐Rhener Equation (9) with the specific volume (𝑣̄), the polymer‐solvent interaction (X1), and the average molecular weight before crosslinks (M_n_) (Table 2).

(9) $$\begin{array}{c} Molecular\;weight\;between\;crosslinks\;\;\left(\frac{1}{M_{c}}\right)\\ =\frac{2}{M_{n}}\;-\frac{\frac{\bar{\nu }}{V_{1}}\;\left[\ln\left(1-v_{2s}\right)+v_{2s}+X_{1}{v_{2s}}^{2}\right]}{v_{2r}\left[{\left(\frac{v_{2s}}{v_{2r}}\right)}^{\frac{1}{3}}-\frac{1}{2}\left(\frac{v_{2s}}{v_{2r}}\right)\right]} \end{array}$$

### Isolation of Human Pre‐Osteoblasts

Human pre‐osteoblasts (hOBs) were isolated from hip explants as previously described.^[^ ^85^ ^]^ The QUT Human Research Ethics Committee (HREC) has provided approval for the primary human osteoblast work: 1400001024. Briefly, trabecular bone was scraped from the explants and cut into small chips, and washed with PBS containing 1% penicillin G and streptomycin (Pen/Strep, Invitrogen) until the solution was clear, removing blood and fat. The bone chips were then transferred to a culture flask with Minimal Essential Medium (MEM‐alpha, Invitrogen) supplemented with 1% Pen/Strep and 10% fetal bovine serum (FBS, ThermoFisher). Cells were incubated at 37 °C, 5% CO_2_, with medium replaced every 3–4 days. A minimal volume of medium was used to keep the bone chips in contact with the culture surface. Cells began to outgrow the bone chips after 7–10 days, and harvesting occurred three times from the chips at 80% confluency. Differentiation ability of the isolated hOBs was confirmed with Alizarin red s staining and Alkaline phosphatase (ALP) activity quantification after 2, 4, and 8 weeks of culturing the hOBs in osteogenic medium.

### Osteogenic Differentiation of Human Osteoblasts

The differentiation (mineralization) of hOBs was induced with osteogenic medium composed of MEM‐alpha medium described above, supplemented with 50 µg mL^−1^ ascorbate‐2‐phosphate (A8960, Sigma–Aldrich), 10 mM β‐glycerophosphate (G9891, Sigma–Aldrich), 100 nM dexamethasone (D2915, Sigma–Aldrich).

### Cell Encapsulation in dECM‐MA and GelMA

hOBs in passages 3–5 were trypsinized (0.25% Trypsin‐EDTA, Gibco, Thermofisher Scientific) and resuspended in 1% (w/v), 2% (w/v) dECM‐MA and 5% (w/v) GelMA hydrogels at a final cell density of 2 × 10^6^ cells mL^−1^. Hydrogels were crosslinked using a 25 µL volume per construct. Encapsulated cells were incubated at 37 °C, 5% CO_2_ in osteogenic medium, which was replaced every 3–4 days.

### Cell Viability

Cell viability was assessed using fluorescein diacetate (FDA, 10 µg mL^−1^ Sigma–Aldrich) and propidium iodide (PI, 5 µg mL^−1^, Sigma–Aldrich) for live/dead staining. Hydrogels were incubated in FDA/PI diluted in PBS at 37 °C for 5 min, then washed in PBS to remove excess stain before imaging. Hydrogels were imaged using a Zeiss Axio Observer 7 equipped with Apotome at 10x magnification. Z‐stack fluorescence images were captured, covering ≈100 µm of the hydrogel (60 stacks of 1.7 µm each). Cell viability was quantified as the percentage of living cells relative to total cells (living and dead cells) using CellProfiler software. Image analysis was performed on three images per hydrogel replicate, with a total of 6–9 images analyzed per condition.

### Alizarin Red S Staining

Mineralization within the hydrogels was qualitatively assessed using Alizarin Red S staining. The hydrogels were rinsed with PBS before fixing the hydrogels with 4% PFA for 1 h. After fixation, the hydrogels were washed twice with ultrapure water, then incubated with 1% (w/v) Alizarin Red S dye (AZR, Sigma, Cat no. A5533) solution for 10 min at room temperature. After staining, excess dye was removed by washing with ultrapure water until the solution clarified, ensuring consistent washes across wells and time points. Stained hydrogels were imaged using a Zeiss Axio Observer 7 microscope equipped with Apotome and a Nikon MacroImager. For semi‐quantitative analysis, Alizarin Red S dye was extracted from the hydrogels using 10% acetic acid. Hydrogels were incubated in acetic acid at room temperature for 30 min, then homogenized using 18G needles and transferred to microcentrifuge tubes. The samples were then heated to 85 °C for 10 min, followed by. cooling on ice for 10 min. After cooling, the samples were centrifuged at 20,000 x g for 15 min, and the supernatant was transferred to a transparent 96‐well plate. To neutralize the extract, 10% (v/v) ammonium hydroxide (NH_4_OH, Sigma–Aldrich) solution was added to the samples, and the absorbance was measured at 405 nm using a CLARIOstar plate reader (BMG Labtech, Germany).

### Alkaline Phosphatase (ALP) Activity

ALP activity was quantified to assess the osteogenic activity of the human osteoblasts. The hydrogels were washed with PBS and transferred to 1.5 mL microcentrifuge tubes before incubation in CelLytic M (C2978, Sigma–Aldrich) for 15 min on a shaker at room temperature. The hydrogels were homogenized using needles and centrifuged at 20 000 x g for 15 min at 4 °C. A dilution series of p‐nitrophenol stock (10 µmol mL^−1^, N7660, Sigma–Aldrich) was prepared, ranging from 200 to 12.5 nmol mL^−1^, in a buffer containing 0.33% (v/v) alkaline buffer (A9226, Sigma–Aldrich) and 0.2% (v/v) Igepal CA‐630 (I3021, Sigma–Aldrich) in ultrapure water. The cell lysates (10% (v/v) were transferred to the 96‐well plate, and 90% (v/v) substrate buffer containing 0.33% (v/v) alkaline buffer and 13.33% (w/v) phosphatase substrate (P4744, Sigma–Aldrich) in ultrapure water was added. The samples and standards were incubated at 37 °C protected from light for 60–90 min until the color changed. The time was recorded, and the reaction was terminated with a stop buffer of 40% (w/v) NaOH in ultrapure water. The absorbance of the samples was measured at 405 nm using a CLARIOstar plate reader (BMG Labtech, Germany).

### Cell Morphology

Cell morphology was assessed using 4',6‐diamidino‐2‐phenylindole (DAPI, 5 µg mL^−1^, cat. no. D1306, Invitrogen, USA) and Alexa Fluor 488 Phalloidin (Phalloidin, 0,165 µM, cat. No. A12379, Invitrogen, USA). The hydrogels were fixed in 4% PFA for 1 h, then blocked overnight in 5% bovine serum albumin (BSA, Sigma–Aldrich, Australia) with 0.1% Triton‐X‐100 in PBS at 4 °C. The hydrogels were then incubated with Phalloidin and DAPI overnight at 4 °C. The hydrogels were imaged using a confocal microscope (FV4000, Olympic) at excision 400 nm (DAPI) and 488 nm (Phalloidin) to capture z‐stack fluorescence images, covering ≈100 µm of the gel (29 stacks of 3.4 µm each). The cell area, major axis length, and eccentricity were determined using a CellProfiler pipeline using CellProfiler 4.2.6 for 2 different hydrogels per biological replicate (*n* = 2) and 3 regions of interest per hydrogel.

### Cell Metabolic Activity

The metabolic activity was measured with a PrestoBlue assay (Thermo Fisher Scientific, Waltham, MA, USA) following the manufacturer's protocol. In short, the medium was removed from the hydrogels and replaced with a solution of 10% (v/v) PrestoBlue in warm media. The hydrogels were incubated at 37 °C, 5% CO_2_ for 45 min before the PrestoBlue media solution and control were aliquoted in triplicate in a clear 96 well plate. The fluorescence was read at 540 nm (ex) and 590 nm (em) using the CLARIOstar plate reader (BMG Labtech, Germany). The results were standardized against the control (PrestoBlue in media without cell‐laden hydrogels).

### µCT Analysis

The mineralization of the hydrogels was quantified using µCT 50 imaging at a source voltage of 55 kV, 72 µA, 8W, a FOV/diameter of 15.2 mm, and a voxel size of 4.4 µm, following previously published methods^[^ ^80^ ^]^ as described above.

### Gene Expression Analysis

Quantitative reverse transcription real‐time polymerase chain reaction (qRT‐PCR) was performed to quantify the response of the hOBs to the different dECM‐MA (5, 10, 20 kPa) and GelMA (5 kPa) hydrogels after 8 weeks of encapsulation as described previously.^[^ ^86^ ^]^ The hydrogels were washed with PBS and transferred to a microcentrifuge tube with TRIzol agent (Thermofisher Scientific, Australia) and were stored at −80 °C. The hydrogels were mechanically disrupted using needles to homogenize the samples and centrifuged for 2 min at 11 000 x g to remove the remaining gel material. The RNA was isolated using the Isolate II RNA Mini Kit (Bioline, Australia) following the manufacturer's instructions. The quality and quantity of the isolated RNA were determined using the Nanodrop OneC spectrophotometer (ThermoFisher Scientific, USA). The isolated RNA (≈100 ng) was reversed transcribed into cDNA using SensiFast cDNA Synthesis Kit (Bioline, Australia) following the manufacturer's protocol. The cDNA was diluted 1:10 in RNase free water and stored at −80 °C until use. qRT‐PCR was performed for multiple osteogenic primers listed in Table 2, using a SYBR Green PCR Master Mix (Applied Biosystems) and ViiA 7 Real‐Time PCR System (life technologies, Australia). The ΔCT values were calculated using the RPL32 housekeeping gene CT value. The fold change of the mRNA levels compared to the housekeeping gene was determined with an adaptation of the ΔΔCT method. The fold change was calculated using the average of the GelMA replicates following the formula:

(10) $$Fold\;change=\frac{2^{-\;\Delta CT\;goi\;sample}}{average\;\left(2^{-\Delta CT\;goi\;control}\right)\;}$$

Where goi is the gene of interest of the sample and the GelMA control group.

The average fold change between the samples and the GelMA control group was calculated by dividing the average fold change by the average fold change of the GelMA control group.

### Immunohistochemistry of Cell‐Laden Hydrogels

The dECM‐MA and GelMA hydrogels were fixed in 4% PFA for 1 h, processed as described, and sectioned into 4 µm slices using the central portion of each hydrogel. The slices were stained for osteocalcin (OC), collagen 1 (hCOL‐1), and DMP‐1 (**Table** **3**) for immunohistochemistry (IHC) following previous standard protocols.^[^ ^14^, ^87^ ^]^

**Table 3:** Antibodies immunohistochemistry.

Marker	Cat. No.	Company	Anti‐Species	Antigen Retrieval	Dilution	Incubation	Color Development
OC	ab133612	Abcam (Cambridge, UK)	Human	Proteinase K, 5 min, RT	1:300	1 h, RT	30 sec
DMP‐1	M176	Takara Bio (USA)	Human, Mouse	Proteinase K, 5 min, RT	1:800	1 h, RT	1 min
hCOL‐1	ab138492	Abcam (Cambridge, UK)	Human	Tris‐EDTA pH 9.0, 5 min, 95°C	1:500	1 h, RT	20 sec

### Statistical Analysis

All data processing and statistical analysis were performed using GraphPad Prism v10.0.2. A Student's *t*‐test with Welch's correction was used for comparing data with 2 conditions, a one‐way ANOVA with Tukey's multiple comparisons for data with three or more conditions, and grouped data was analyzed using a two‐way ANOVA with Sidak's multiple comparisons. A *p*‐value below 0.05 was considered statistically significant. All graphs show the data as the mean ± the standard deviation (SD).

### Ethical Statement

The QUT Human Research Ethics Committee (HREC) has provided approval for the primary human osteoblast work: 1400001024. Written informed consent was obtained from all human participants involved in providing primary cells for research purposes prior to cell isolation.

## Conflict of Interest

C.M. is the Chief Executive Officer, a Co‐Founder, Shareholder, and Executive Director of Gelomics Pty Ltd. D.W.H. is a Co‐Founder, Shareholder, and member of the Scientific Advisory Board of Gelomics Pty Ltd. L.H. is employed by Gelomics Pty Ltd. Both L.H. and M.D. are PhD candidates co‐supervised by Gelomics Pty Ltd. All other authors declare no competing interests.

## Author Contributions

M.D. dealt with conceptualization, methodology, formal analysis, investigation, data curation, visualization, Project administration, and wrote the original draft. L.H. assisted with methodology and investigation, and wrote, reviewed, and edited the final manuscript. A.R. and D.W.H. contributed supervision, as well as wrote, reviewed, and edited the final manuscript. C.M. carried out conceptualization, methodology, investigation, resources, supervision, project administration, funding acquisition, and wrote, reviewed, and edited the final manuscript. J.M. carried out conceptualization, methodology, investigation, resources, supervision, project administration, funding acquisition, and wrote, reviewed, and edited the final manuscript. All authors read and reviewed the manuscript and agreed on the final curated version of the manuscript.

## Supporting information



Supporting Information

## Data Availability

The data that support the findings of this study are available from the corresponding author upon reasonable request.

## References

[1] X. Lin , S. Patil , Y. G. Gao , A. Qian , Front. Pharmacol. 2020, 11, 757. 32528290 10.3389/fphar.2020.00757 PMC7264100

[2] D. Keun Han , Y. Gao , X. Zhang , H. Zhou , Pharmaceutics 2023, 15, 2405. 37896165 10.3390/pharmaceutics15102405 PMC10609742

[3] G. Li , D. Zhou , S. Sheng , Q. Lin , Y. Jing , X. Ren , J. Su , Chem. Eng. J. 2024, 499, 156554.

[4] S. Yue , H. He , B. Li , T. Hou , Nanomaterials 2020, 10, 1511. 32752105 10.3390/nano10081511 PMC7466535

[5] X. Xue , Y. Hu , Y. Deng , J. Su , Adv. Funct. Mater. 2021, 31, 2009432.

[6] G. S. Hussey , J. L. Dziki , S. F. Badylak , Nat. Rev. Mater. 2018, 3, 159.

[7] H. Amirazad , M. Dadashpour , N. Zarghami , J. Biol. Eng. 2022, 16, 1. 34986859 10.1186/s13036-021-00282-5 PMC8734306

[8] X. Zhang , X. Chen , H. Hong , R. Hu , J. Liu , C. Liu , Bioact Mater 2022, 10, 15. 34901526 10.1016/j.bioactmat.2021.09.014 PMC8637010

[9] G. Chen , Y. Lv , Methods in Mol. Biol. 2018, 1577, 239. 28770492 10.1007/7651_2017_50

[10] M. J. Sawkins , W. Bowen , P. Dhadda , H. Markides , L. E. Sidney , A. J. Taylor , F. R. A. J. Rose , S. F. Badylak , K. M. Shakesheff , L. J. White , Acta Biomater. 2013, 9, 7865. 23624219 10.1016/j.actbio.2013.04.029 PMC3711237

[11] N. Alom , H. Peto , G. R. Kirkham , K. M. Shakesheff , L. J. White , J. Biomed. Mater. Res. B Appl. Biomater. 2018, 106, 900. 28429412 10.1002/jbm.b.33894

[12] Y.‐H. Kim , G. Cidonio , J. M. Kanczler , R. C. Oreffo , J. I. Dawson , Bioact. Mater. 2025, 43, 114. 39376928 10.1016/j.bioactmat.2024.09.007 PMC11456876

[13] S. P. Parthiban , A. Athirasala , A. Tahayeri , R. Abdelmoniem , A. George , L. E. Bertassoni , Biofabrication 2021, 13, 035031. 10.1088/1758-5090/abb11f PMC9059555 35130535

[14] A. Bessot , F. M. Savi , J. Gunter , J. Mendhi , S. Amini , D. Waugh , J. Mcgovern , D. W. Hutmacher , N. Bock , Adv. Healthcare Mater. 2025, 14, 2401939. 10.1002/adhm.202401939 PMC11729988 39444080

[15] S. S. Lee , X. Du , I. Kim , S. J. Ferguson , Matter 2022, 5, 2722.

[16] G. Hannink , J. J. C. Arts , Injury 2011, 42, S22. 10.1016/j.injury.2011.06.008 21714966

[17] K. V. Greco , L. Francis , M. Somasundaram , G. Greco , N. R. English , J. A. Roether , A. R. Boccaccini , P. Sibbons , T. Ansari , J. Biomater. Appl. 2015, 30, 239. 25855682 10.1177/0885328215578638

[18] A. Emami , T. Talaei‐Khozani , Z. Vojdani , N. Zarei fard , J. Biomed. Mater. Res., Part B 2021, 109, 19. 10.1002/jbm.b.34677 32627321

[19] C. Gardin , S. Ricci , L. Ferroni , R. Guazzo , L. Sbricoli , G. De Benedictis , L. Finotti , M. Isola , E. Bressan , B. Zavan , PLoS One 2015, 10, 0132344. 10.1371/journal.pone.0132344 PMC4507977 26191793

[20] J. Lee , J. Hong , W. J. Kim , G. H. Kim , Carbohydr. Polym. 2020, 250, 116914. 33049834 10.1016/j.carbpol.2020.116914

[21] Z. Nie , X. Wang , L. Ren , Y. Kang , Regener. Med. 2020, 15, 1519. 10.2217/rme-2019-0125 PMC8977946 32441554

[22] R. Menezes , R. Vincent , L. Osorno , P. Hu , T. L. Arinzeh , Acta Biomater. 2023, 163, 210. 36182056 10.1016/j.actbio.2022.09.064 PMC10043054

[23] T. Guo , X. Yuan , X. Li , Y. Liu , J. Zhou , J. Dent. Sci. 2023, 18, 135. 36643246 10.1016/j.jds.2022.06.020 PMC9831827

[24] E. A. Calle , R. C. Hill , K. L. Leiby , A. V. Le , A. L. Gard , J. A. Madri , K. C. Hansen , L. E. Niklason , Acta Biomater. 2016, 46, 91. 27693690 10.1016/j.actbio.2016.09.043 PMC5451113

[25] U. Rende , S. B. Ahn , S. Adhikari , E. S. X. Moh , C. A. Pollock , S. Saad , A. Guller , Int. J. Mol. Sci. 2023, 24, 2827. 36769148 10.3390/ijms24032827 PMC9917693

[26] R. Gaetani , S. Aude , L. L. Demaddalena , H. Strassle , M. Dzieciatkowska , M. Wortham , R. H. F. Bender , K. V. Nguyen‐Ngoc , G. W. Schmid‐Schöenbein , S. C. George , C. W. W. Hughes , M. Sander , K. C. Hansen , K. L. Christman , Tissue Eng., Part C 2018, 24, 697. 10.1089/ten.tec.2018.0180 PMC6306687 30398401

[27] G. Major , J. Simcock , A. Kumar , T. Kleffmann , T. B. F. Woodfield , K. S. Lim , Adv. Biol. 2024, 8, 2300448. 10.1002/adbi.202300448 37953659

[28] E. Sonpho , F. G. Mann , M. Levy , E. J. Ross , C. Guerrero‐Hernández , L. Florens , A. Saraf , V. Doddihal , P. Ounjai , A. S. Alvarado , Mol. Cell. Proteomics 2021, 20, 100137. 34416386 10.1016/j.mcpro.2021.100137 PMC8503668

[29] V. Selvaraj , S. Sekaran , A. Dhanasekaran , S. Warrier , Differentiation 2024, 136, 100757. 38437764 10.1016/j.diff.2024.100757

[30] S. P. Boudko , N. Danylevych , B. G. Hudson , V. K. Pedchenko , Methods Cell Biol. 2017, 143, 171. 29310777 10.1016/bs.mcb.2017.08.010 PMC5828530

[31] X. Shao , C. D. Gomez , N. Kapoor , J. M. Considine , C. Grams , Y. Gao , A. Naba , Nucleic Acids Res. 2023, 51, D1519. 36399478 10.1093/nar/gkac1009 PMC9825471

[32] K. J. Hamill , K. Kligys , S. B. Hopkinson , J. C. R. Jones , J. Cell Sci. 2009, 122, 4409. 19955338 10.1242/jcs.041095 PMC2787456

[33] E. Wei , M. Hu , L. Wu , X. Pan , Q. Zhu , H. Liu , Y. Liu , Stem Cell Res. Ther. 2024, 15, 156. 38816830 10.1186/s13287-024-03761-w PMC11140988

[34] I. Pepelanova , K. Kruppa , T. Scheper , A. Lavrentieva , Bioengineering 2018, 5, 55. 30022000 10.3390/bioengineering5030055 PMC6165498

[35] J. W. Nichol , S. T. Koshy , H. Bae , C. M. Hwang , S. Yamanlar , A. Khademhosseini , Biomaterials 2010, 31, 5536. 20417964 10.1016/j.biomaterials.2010.03.064 PMC2878615

[36] M. Sun , G. Chi , J. Xu , Y. Tan , J. Xu , S. Lv , Z. Xu , Y. Xia , L. Li , Y. Li , Stem Cell Res. Ther. 2018, 9, 52. 29490668 10.1186/s13287-018-0798-0 PMC5831741

[37] A. Bessot , J. Gunter , D. Waugh , J. A. Clements , D. W. Hutmacher , J. Mcgovern , N. Bock , Adv. Healthcare Mater. 2023, 12, 2201701. 10.1002/adhm.202201701 PMC11469108 36708740

[38] T. Luo , B. Tan , L. Zhu , Y. Wang , J. Liao , Front Bioeng. Biotechnol. 2022, 10, 817391. 35145958 10.3389/fbioe.2022.817391 PMC8822157

[39] X. Hao , S. Miao , Z. Li , T. Wang , B. Xue , J. Chen , C. Xian , L. Bi , Mater. Des. 2023, 227, 111729.

[40] T. A. Franz‐Odendaal , B. K. Hall , P. E. Witten , Dev. Dyn. 2006, 235, 176. 16258960 10.1002/dvdy.20603

[41] N. Sawa , H. Fujimoto , Y. Sawa , J. Yamashita , Sci. Rep. 2019, 9, 1. 31554848 10.1038/s41598-019-50236-7 PMC6761144

[42] J. Kim , T. Adachi , Front Bioeng. Biotechnol. 2019, 7, 288. 31709248 10.3389/fbioe.2019.00288 PMC6819367

[43] G. Nasello , P. Alamán‐Díez , J. Schiavi , M. Á. Pérez , L. McNamara , J. M. García‐Aznar , Front. Bioeng. Biotechnol. 2020, 8, 518007. 10.3389/fbioe.2020.00336 PMC7193048 32391343

[44] F. G. F. Tresguerres , J. Torres , J. López‐Quiles , G. Hernández , J. A. Vega , I. F. Tresguerres , Ann. Anat. 2020, 227, 151422. 31563568 10.1016/j.aanat.2019.151422

[45] D. Lopes , C. Martins‐Cruz , M. B. Oliveira , J. F. Mano , Biomaterials 2018, 185, 240. 30261426 10.1016/j.biomaterials.2018.09.028 PMC6445367

[46] A. Ravichandran , B. Murekatete , D. Moedder , C. Meinert , L. J. Bray , Sci. Rep. 2021, 11, 1. 34330947 10.1038/s41598-021-94990-z PMC8324893

[47] P. M. Crapo , T. W. Gilbert , S. F. Badylak , Biomaterials 2011, 32, 3233. 21296410 10.1016/j.biomaterials.2011.01.057 PMC3084613

[48] J. Fernández‐Pérez , M. Ahearne , Sci. Rep. 2019, 9, 1. 31624357 10.1038/s41598-019-49575-2 PMC6797749

[49] C. Mueller , M. Gambarotti , S. Benini , P. Picci , A. Righi , M. Stevanin , S. Hombach‐Klonisch , D. Henderson , L. Liotta , V. Espina , Lab. Invest. 2019, 99, 708. 30659273 10.1038/s41374-018-0168-7 PMC10752433

[50] M. Fontcuberta‐Rigo , M. Nakamura , P. Puigbò , Bone Res. 2023, 11, 1. 37580331 10.1038/s41413-023-00281-w PMC10425349

[51] D. Niu , X. Ma , T. Yuan , Y. Niu , Y. Xu , Z. Sun , Y. Ping , W. Li , J. Zhang , T. Wang , G. M. Church , Adv. Drug. Deliv. Rev. 2021, 168, 229. 32275950 10.1016/j.addr.2020.04.001

[52] J. Suñer‐Carbó , R. Hayam , S. Hamias , M. Skitel Moshe , T. Davidov , F.‐C. Yen , L. Baruch , M. Machluf , Gels 2025, 11, 173. 40136879 10.3390/gels11030173 PMC11942433

[53] F. Obregon‐Miano , A. Fathi , C. Rathsam , I. Sandoval , F. Deheghani , A. Spahr , J. Mater. Sci. Mater. Med. 2020, 31, 1. 10.1007/s10856-019-6354-3 31989310

[54] S. Li , R. Deng , T. Forouzanfar , G. Wu , D. Quan , M. Zhou , Gels 2022, 8, 294. 35621593 10.3390/gels8050294 PMC9140703

[55] R. D. Ventura , A. R. Padalhin , B. Kim , M. K. Park , B. T. Lee , Mater. Sci. Eng., C 2020, 110, 110663. 10.1016/j.msec.2020.110663 32204091

[56] D. Gothard , E. L. Smith , J. M. Kanczler , C. R. Black , J. A. Wells , C. A. Roberts , L. J. White , O. Qutachi , H. Peto , H. Rashidi , L. Rojo , M. M. Stevens , A. J. El Haj , F. R. A. J. Rose , K. M. Shakesheff , R. O. C. Oreffo , PLoS One 2015, 10, 0145080. 10.1371/journal.pone.0145080 PMC4684226 26675008

[57] Y.‐H. Kim , G. Cidonio , J. M. Kanczler , R. O. Oreffo , J. I. Dawson , Bioact. Mater. 2024, 43, 114. 39376928 10.1016/j.bioactmat.2024.09.007 PMC11456876

[58] X. Zhang , S. Zhou , Y. Zhan , Z. Mei , A. Qian , Y. Yuan , X. Zhang , T. Fu , S. Ma , J. Li , Mater. Today Bio 2024, 25, 100990. 10.1016/j.mtbio.2024.100990 PMC10873736 38371466

[59] N. Huebsch , P. R. Arany , A. S. Mao , D. Shvartsman , O. A. Ali , S. A. Bencherif , J. Rivera‐Feliciano , D. J. Mooney , Nat. Mater. 2010, 9, 518. 20418863 10.1038/nmat2732 PMC2919753

[60] O. Chaudhuri , L. Gu , D. Klumpers , M. Darnell , S. A. Bencherif , J. C. Weaver , N. Huebsch , H. P. Lee , E. Lippens , G. N. Duda , D. J. Mooney , Nat. Mater. 2015, 15, 326. 26618884 10.1038/nmat4489 PMC4767627

[61] A. K. Jha , W. M. Jackson , K. E. Healy , PLoS One 2014, 9, 98640. 10.1371/journal.pone.0098640 PMC4060996 24937602

[62] L. Oliver‐Cervelló , H. Martin‐Gómez , C. Gonzalez‐Garcia , M. Salmeron‐Sanchez , M. P. Ginebra , C. Mas‐Moruno , Front. Bioeng. Biotechnol. 2023, 11, 1192436. 37324414 10.3389/fbioe.2023.1192436 PMC10267393

[63] B. Trappmann , J. E. Gautrot , J. T. Connelly , D. G. T. Strange , Y. Li , M. L. Oyen , M. A. Cohen Stuart , H. Boehm , B. Li , V. Vogel , J. P. Spatz , F. M. Watt , W. T. S. Huck , Nat. Mater. 2012, 11, 642. 22635042 10.1038/nmat3339

[64] N. Huebsch , E. Lippens , K. Lee , M. Mehta , S. T. Koshy , M. C. Darnell , R. M. Desai , C. M. Madl , M. Xu , X. Zhao , O. Chaudhuri , C. Verbeke , W. S. Kim , K. Alim , A. Mammoto , D. E. Ingber , G. N. Duda , D. J. Mooney , Nat. Mater. 2015, 14, 1269. 26366848 10.1038/nmat4407 PMC4654683

[65] K. T. Campbell , K. Wysoczynski , D. J. Hadley , E. A. Silva , ACS Biomater. Sci. Eng. 2019, 6, 308. 33313390 10.1021/acsbiomaterials.9b01520 PMC7725240

[66] K. J. De France , F. Xu , T. Hoare , Adv. Healthcare Mater. 2018, 7, 1700927. 10.1002/adhm.201700927 29195022

[67] V. Karageorgiou , D. Kaplan , Biomaterials 2005, 26, 5474. 15860204 10.1016/j.biomaterials.2005.02.002

[68] G. Thrivikraman , A. Athirasala , R. Gordon , L. Zhang , R. Bergan , D. R. Keene , J. M. Jones , H. Xie , Z. Chen , J. Tao , B. Wingender , L. Gower , J. L. Ferracane , L. E. Bertassoni , Nat. Commun. 2019, 10, 1. 31388010 10.1038/s41467-019-11455-8 PMC6684598

[69] B. Zhou , X. Jiang , X. Zhou , W. Tan , H. Luo , S. Lei , Y. Yang , Biomater. Res. 2023, 27, 1. 37715230 10.1186/s40824-023-00422-6 PMC10504735

[70] Y. Zhu , X. Yu , H. Liu , J. Li , M. Gholipourmalekabadi , K. Lin , C. Yuan , P. Wang , Bioact. Mater. 2024, 38, 346. 38764449 10.1016/j.bioactmat.2024.04.032 PMC11101688

[71] A. A. Golebiowska , J. T. Intravaia , V. M. Sathe , S. G. Kumbar , S. P. Nukavarapu , Bioact. Mater. 2024, 32, 98. 37927899 10.1016/j.bioactmat.2023.09.017 PMC10622743

[72] N. A. Bock , T. Shokoohmand , J. Kryza , J. Röhl , P. A. Meijer , C. C. Tran , J. A. Nelson , D. W. H. Clements , Bone Res. 2019, 7, 1. 31044095 10.1038/s41413-019-0049-8 PMC6486620

[73] H. Cao , L. Duan , Y. Zhang , J. Cao , K. Zhang , Signal Transduction Targeted Ther. 2021, 6, 1. 10.1038/s41392-021-00830-x PMC8674418 34916490

[74] J. Kort‐Mascort , S. Flores‐Torres , O. Peza‐Chavez , J. H. Jang , L. A. Pardo , S. D. Tran , J. Kinsella , Biomater. Sci. 2023, 11, 400. 36484344 10.1039/d2bm01273a

[75] Z. Chen , J. Wang , R. K. Kankala , M. Jiang , L. Long , W. Li , L. Zou , A. Chen , Y. Liu , Mater. Today Bio 2024, 29, 101280. 10.1016/j.mtbio.2024.101280 PMC11470555 39399243

[76] L. A. Milton , J. W. Davern , L. Hipwood , J. C. S. Chaves , J. McGovern , D. Broszczak , D. W. Hutmacher , C. Meinert , Y. C. Toh , Acta Biomater. 2024, 185, 144. 38960110 10.1016/j.actbio.2024.06.037

[77] R. Curvello , V. Kast , P. Ordóñez‐Morán , A. Mata , D. Loessner , Nat. Rev. Mater. 2023, 8, 314.

[78] J. Paulos , D. G. Poitout , Bone Tumors: Diagnosis and Therapy Today, Springer, London 2023, 199.

[79] J. Cui , D. Dean , F. J. Hornicek , Z. Chen , Z. Duan , J. Exp. Clinic. Cancer Res. 2020, 39, 1. 10.1186/s13046-020-01685-w PMC7650219 32887645

[80] F. M. Savi , G. I. Brierly , J. Baldwin , C. Theodoropoulos , M. A. Woodruff , J. Histochem. Cytochem. 2017, 65, 705. 28958188 10.1369/0022155417733708 PMC5714097

[81] C. Meinert , C. Theodoropoulos , T. J. Klein , D. W. Hutmacher , D. Loessner , Methods Mol. Biol. 2018, 1786, 175. 29786793 10.1007/978-1-4939-7845-8_10

[82] D. Loessner , C. Meinert , E. Kaemmerer , L. C. Martine , K. Yue , P. A. Levett , T. J. Klein , F. P. W. Melchels , A. Khademhosseini , D. W. Hutmacher , Nat. Protoc. 2016, 11, 727. 26985572 10.1038/nprot.2016.037

[83] N. A. Peppas , E. W. Merrill , J. Appl. Polym. Sci. 1977, 21, 1763.

[84] M. Vigata , C. D. O'connell , S. Cometta , D. W. Hutmacher , C. Meinert , N. Bock , Polymers 2021, 13, 3960. 34833259 10.3390/polym13223960 PMC8618379

[85] J. C. Reichert , V. M. C. Quent , L. J. Burke , S. H. Stansfield , J. A. Clements , D. W. Hutmacher , Biomaterials 2010, 31, 7928. 20688384 10.1016/j.biomaterials.2010.06.055

[86] A. Ravichandran , C. Meinert , O. Bas , D. W. Hutmacher , N. Bock , Mater. Sci. Eng., C 2021, 128, 112313. 10.1016/j.msec.2021.112313 34474864

[87] M. Laubach , B. Herath , N. Bock , S. Suresh , S. Saifzadeh , B. L. Dargaville , J. McGovern , M. L. Wille , D. W. Hutmacher , F. M. Savi , Front. Bioeng. Biotechnol. 2023, 11, 1272348. 37860627 10.3389/fbioe.2023.1272348 PMC10584154

