# Supplementary material for: Bone‐Derived dECM Hydrogels Support Tunable Microenvironments for In Vitro Osteogenic Differentiation
Source: Adv Healthc Mater. 2025 Oct 2;15(1):2501350. doi: 10.1002/adhm.202501350 (PMC12790313; doi:10.1002/adhm.202501350)
Supplement: Supplementary file 1 — Supporting Information [file ADHM-15-0-s001.docx]

Supporting Information

Bone-derived dECM hydrogels support tunable microenvironments for in vitro osteogenic differentiation

*Minne Dekker a c d e, Luke Hipwood a c d e , Akhilandeshwari Ravichandran b c, Dietmar W. Hutmacher d e f h, Christoph Meinert d* and Jacqui McGovern a c e g h**


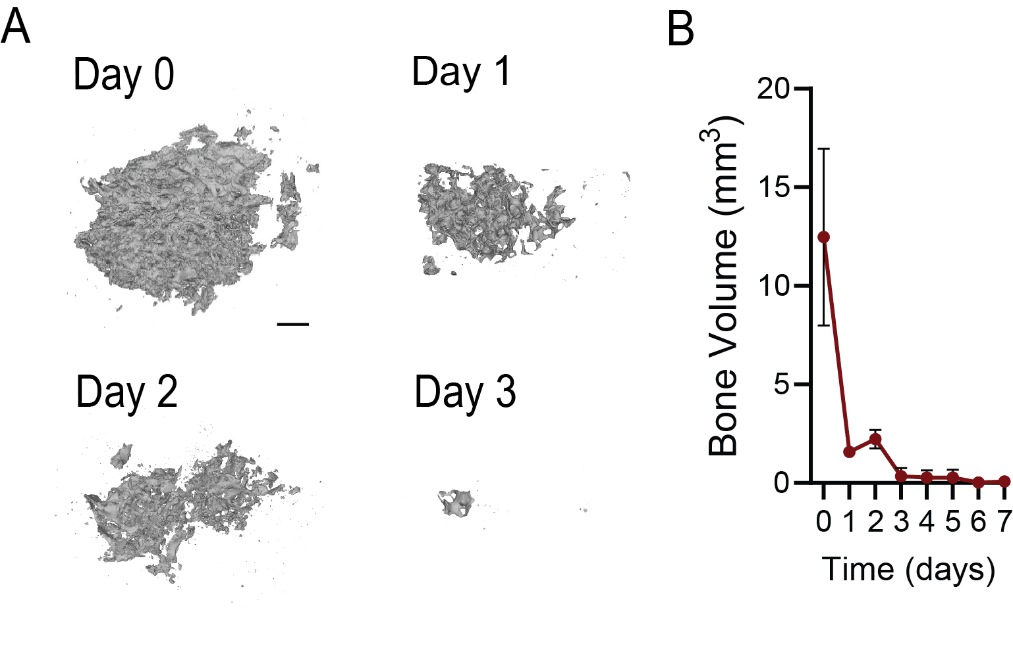


**Figure S1.** Demineralization of porcine bone tissue**.** A) Representative µCT images of bone fragments from day 0 to day 3 of demineralization using 10% w/v EDTA. Scalebar = 1 mm. B) Mineralized bone volume calculated from the µCT images for 2 replicates of representative samples over time. n=2.


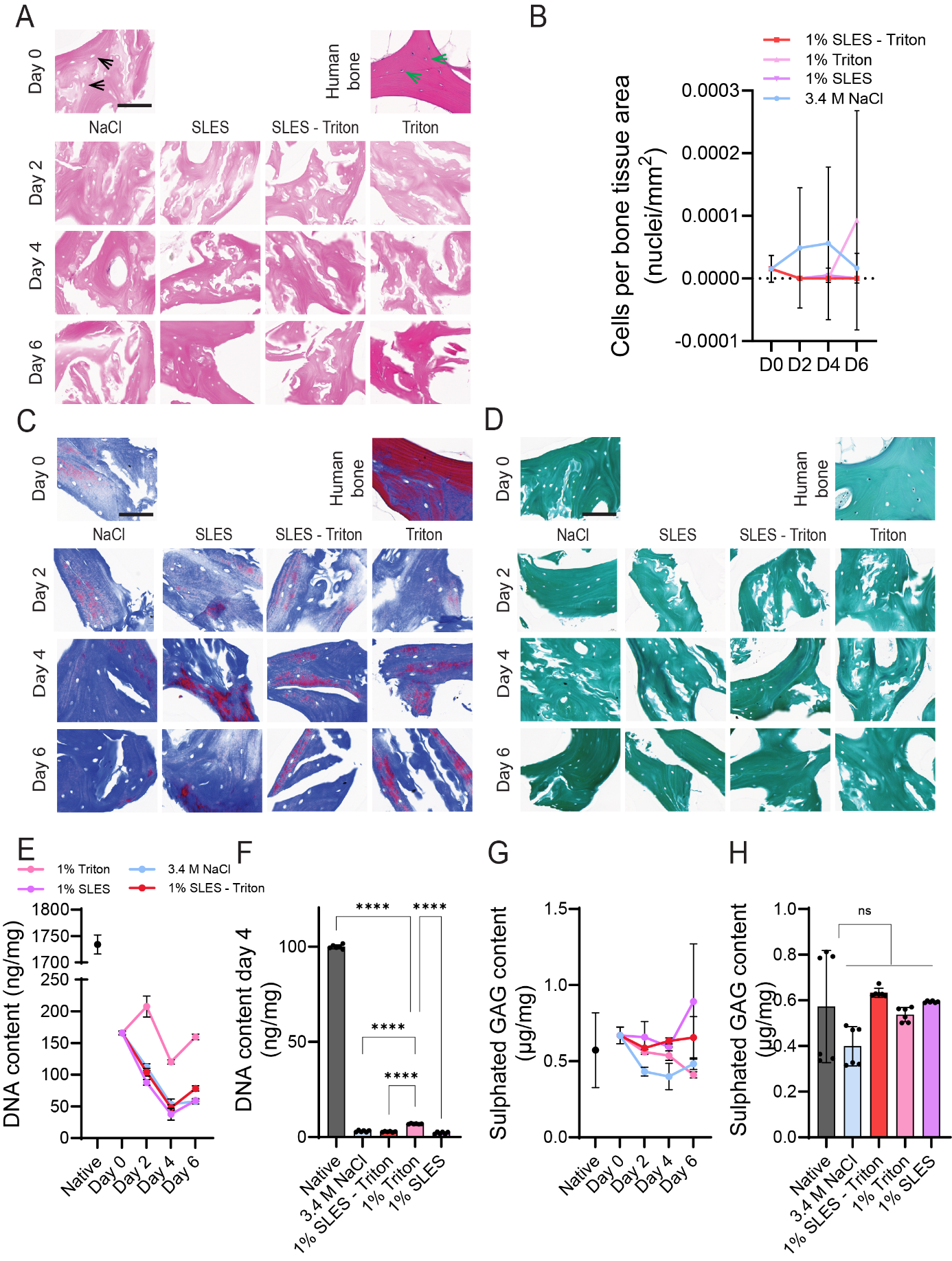


**Figure S2**. Effect of decellularization method on the DNA and GAG content in decellularized bone tissue. A) Hematoxylin and Eosin staining of 4 µm sections. The green arrows show the location of absent cells, while the green arrows show the location of cells indicated in purple present in the control. B) Quantification of the number of nuclei per bone tissue for the four different decellularization methods. C) Masson’s and Trichrome staining of 4 µm sections. D) Safranin O staining of 4 µm sections. For all A, B, D) the scalebar = 100 µm and the magnification is 40x. The control is human bone tissue. Day 0 zero is after demineralization. E) DNA content (ng.mg^-1^ wet tissue weight) using four decellularization methods: 3.4 M NaCl (blue), a sequential application of 1% SLES for three days followed by 1% Triton-X-100 for three days (red), 1% Triton-X-100 (pink), and 1% SLES (lilac) compared to native porcine bone tissue. F) Percentage of DNA removal relative to native tissue on day 4 post-demineralization, with native bone tissue serving as a control. Statistical analysis was performed using an ordinary one-way ANOVA, with significant findings indicated by **** p < 0.0001. G) Chondroitin Sulfate content (µg.mg^-1^ wet tissue weight) for each of the four decellularization methods. H) Chondroitin sulfate content on day 4 post demineralization for all four decellularization methods. No significant differences were observed when compared to the native porcine bone tissue, as determined by an ordinary one-way ANOVA test. A-D) Data represent mean ± SD for six replicates (n=2, 2 biological and 3 technical).


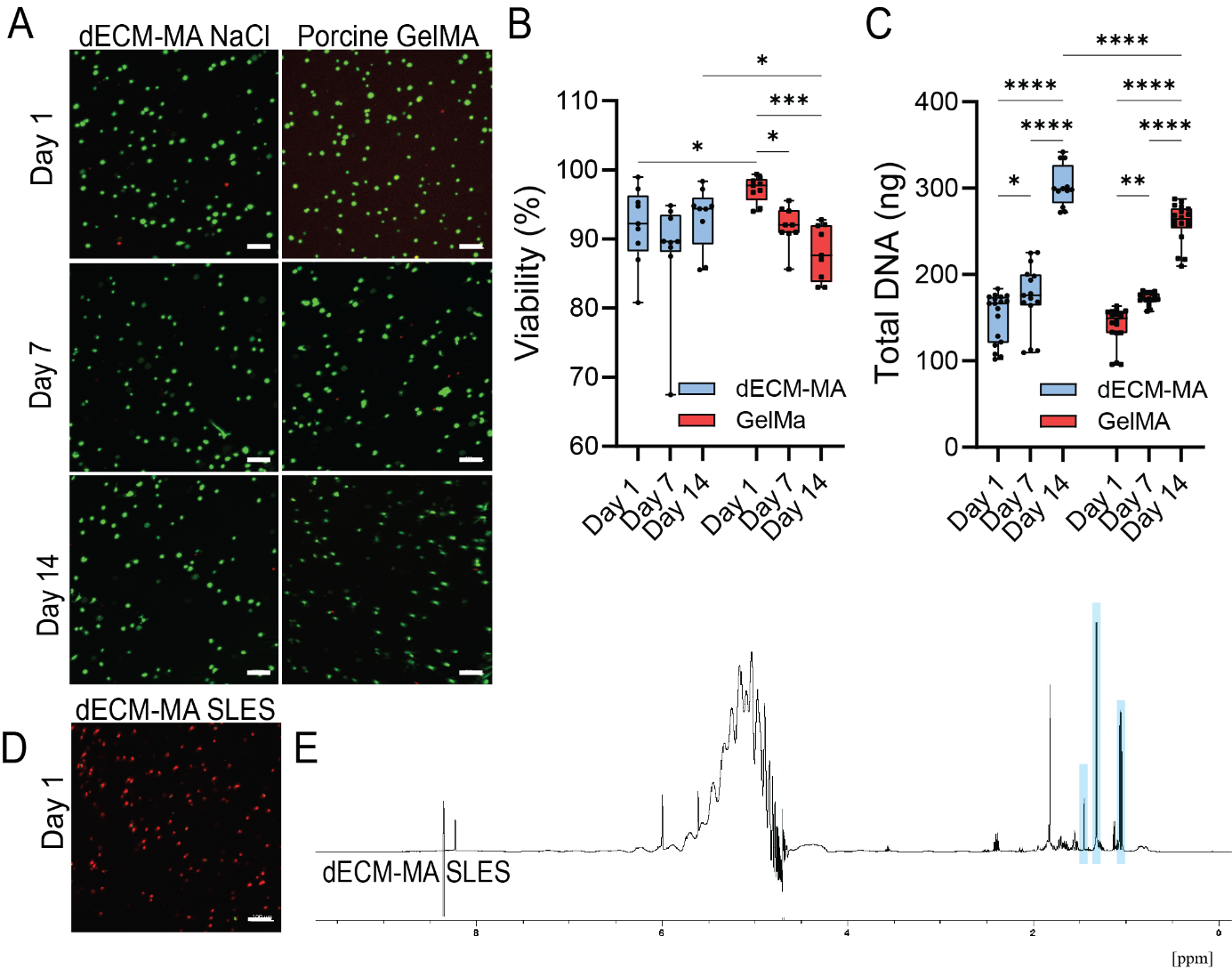


**Figure S3**. Cell viability of the dECM-MA gels decellularized with 1% SLES or 3.4 M NaCl compared to GelMA. A) Live/Dead staining using FDA (green, live cells) and in PI (red, dead cells) of the hOBs encapsulated in dECM-MA decellularized with NaCl and GelMA using 1x10^6^ cells.ml^-1^ on day 1, 7, and 14. Scalebar = 100 µm. B) Quantified viability using CellProfiler software analyzing 3 regions of interest of 3 gels per timepoint (n=9) C) Total DNA content per gel determined using PicoGreen assay. All data represents the mean +/- the SD for 9 replicates. (n=18, 6 biological replicates, 3 technical replicates). Statistical analysis was performed using a two-way ANOVA, with significant findings indicated by * p< 0.05, ** p< 0.01, *** p= 0.0001, **** p<0.0001. D) Live/Dead staining using FDA (green, live cells) and in PI (red, dead cells) of the hOBs encapsulated in dECM-MA decellularized with SLES and GelMA using 1x10^6^ cells.ml^-1^ on day 1, 7, and 14. Scalebar = 100 µm. E) The ^1^H-NMR spectrum of the dECM-MA SLES indicating in blue remnants of SLES present in the dECM-MA, suggesting the non-cytocompatibility of the dECM-MA decellularized with SLES.


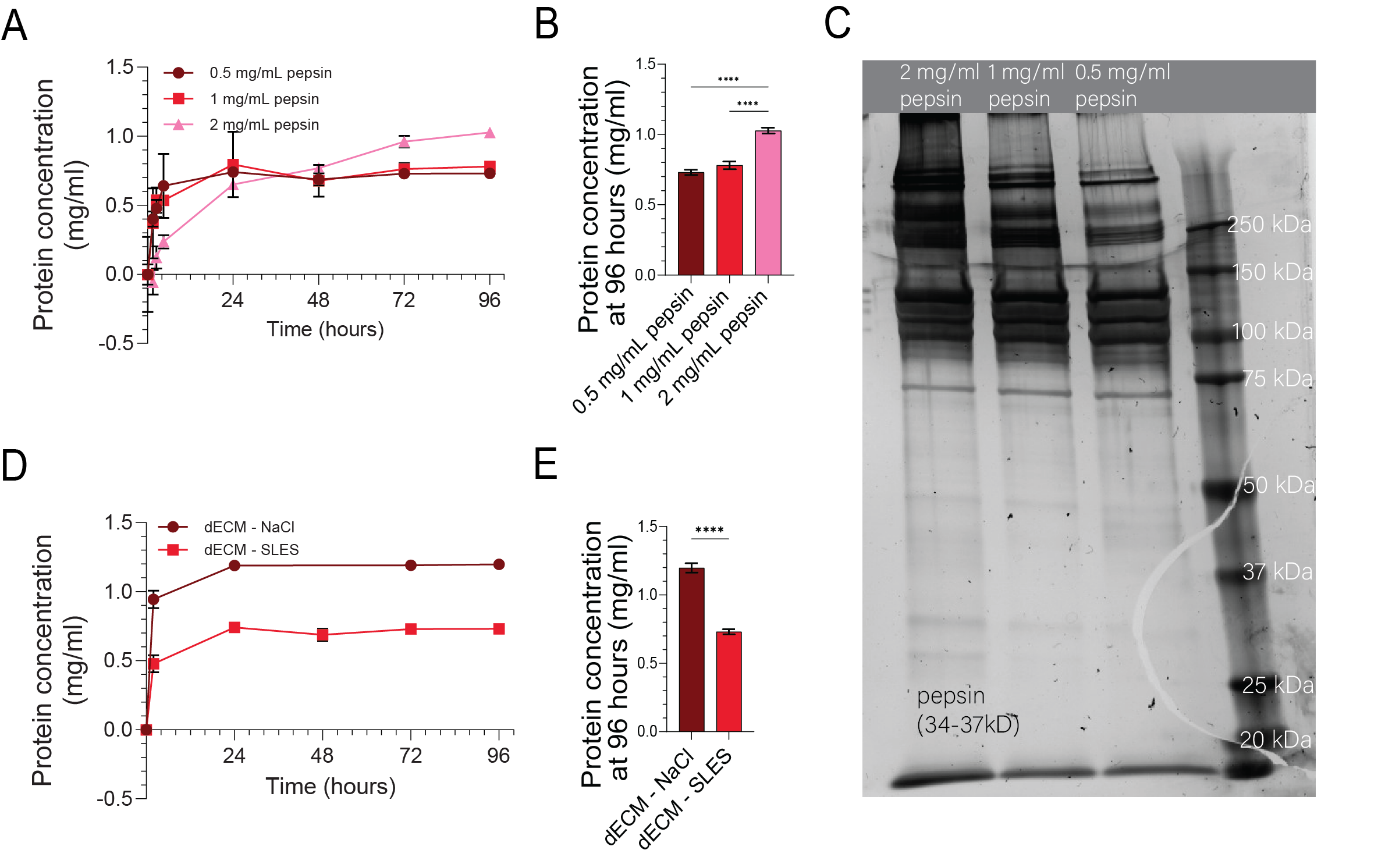


**Figure S4.** Solubilization of dECM using pepsin digestion**.** A) Increase in dissolved dECM protein concentration (mg.ml^-1^) over time comparing different concentrations (0.5, 1, and 2 mg.ml^-1^) of pepsin in 0.01 M HCl. B) Protein concentration (mg.ml^-1^) at 96 hours comparing different concentrations (0.5, 1, and 2 mg.ml^-1^) of pepsin in 0.01 M HCl. Ordinary one-way ANOVA, ****p<0.0001. C) SDS page gel of the solubilized dECM after 96 hours comparing different concentrations (0.5, 1, and 2 mg.ml^-1^) of pepsin in 0.01 M HCl. D) Protein concentration of digested dECM decellularized with NaCl and SLES using 0.5 mg.ml^-1^pepsin in 0.01 M HCl over time. E) Protein concentration of digested dECM decellularized with NaCl and SLES using 0.5 mg.ml^-1^ pepsin in 0.01 M HCl at 96 hours. Unpaired t test, ****p<0.0001. The data represents the mean +/- the standard deviation for six replicates. (n=6, technical replicates).

**Table S1.** Fold changes abundance matrisome proteins comparing native porcine tissue and porcine dECM.

| Protein | Abundance ratio  [log 2] | Abundance ratio adjusted p-value | Matrisome category |
| --- | --- | --- | --- |
| COL10A1 | 3.3 | 1.83E-02 | Collagens |
| COL11A1 | 0.41 | 7.90E-01 | Collagens |
| COL11A2 | 0.83 | 6.74E-01 | Collagens |
| COL12A1 | 2.51 | 1.81E-01 | Collagens |
| COL1A1 | 1.89 | 3.34E-01 | Collagens |
| COL1A2 | 2.56 | 1.70E-01 | Collagens |
| COL27A1 | -0.15 | 9.24E-01 | Collagens |
| COL2A1 | -1.19 | 9.49E-01 | Collagens |
| COL3A1 | -0.31 | 9.00E-01 | Collagens |
| COL4A1 | -9.97 | 3.78E-17 | Collagens |
| COL4A3 | 0.44 | 7.17E-01 | Collagens |
| COL4A6 | -9.97 | 3.78E-17 | Collagens |
| COL5A1 | 2.63 | 1.56E-01 | Collagens |
| COL6A1 | 1.51 | 4.55E-01 | Collagens |
| COL6A2 | 1.57 | 4.36E-01 | Collagens |
| AGRN | 0.98 | 5.13E-01 | ECM Glycoproteins |
| COMP | 0.71 | 7.11E-01 | ECM Glycoproteins |
| EMILIN1 | 1.52 | 4.50E-01 | ECM Glycoproteins |
| FBLN5 | 0.88 | 6.55E-01 | ECM Glycoproteins |
| FBN1 | 1.03 | 6.10E-01 | ECM Glycoproteins |
| FN1 | 1.21 | 5.55E-01 | ECM Glycoproteins |
| LAMA3 | 1.01 | 4.41E-01 | ECM Glycoproteins |
| LAMA4 | 0.08 | 8.57E-01 | ECM Glycoproteins |
| LAMB2 | 0.99 | 6.22E-01 | ECM Glycoproteins |
| LAMC1 | 0.45 | 7.79E-01 | ECM Glycoproteins |
| LAMC2 | -9.97 | 3.78E-17 | ECM Glycoproteins |
| MATN2 | 2.12 | 1.39E-01 | ECM Glycoproteins |
| MFAP4 | 1.56 | 2.49E-01 | ECM Glycoproteins |
| MMRN2 | -9.97 | 3.78E-17 | ECM Glycoproteins |
| SPON1 | 0.66 | 5.91E-01 | ECM Glycoproteins |
| TGFBI | 1.63 | 4.17E-01 | ECM Glycoproteins |
| THBS1 | -1.46 | 9.03E-01 | ECM Glycoproteins |
| THBS4 | -9.97 | 3.78E-17 | ECM Glycoproteins |
| TNN | -1.5 | 8.96E-01 | ECM Glycoproteins |
| TNXB | 0.81 | 4.86E-01 | ECM Glycoproteins |
| VIT | 3.36 | 5.82E-02 | ECM Glycoproteins |
| VWA1 | 2.06 | 9.75E-02 | ECM Glycoproteins |
| ACAN | 1.05 | 6.06E-01 | proteoglycans |
| BGN | 1.87 | 3.38E-01 | proteoglycans |
| DCN | 2.43 | 1.93E-01 | proteoglycans |
| EPYC | 0.25 | 7.99E-01 | proteoglycans |
| HAPLN1 | 2.08 | 2.79E-01 | proteoglycans |
| HSPG2 | 1.72 | 3.88E-01 | proteoglycans |
| LUM | 2.01 | 2.99E-01 | proteoglycans |
| OGN | 1.27 | 5.38E-01 | proteoglycans |
| VCAN | -0.14 | 9.00E-01 | proteoglycans |
| ADAMTS2 | 2.58 | 4.41E-02 | ECM regulators |
| ALPL | 1.2 | 5.55E-01 | ECM regulators |
| HTRA1 | 3.82 | 2.84E-02 | ECM regulators |
| LOX | 5.49 | 1.14E-03 | ECM regulators |
| LOXL2 | 1.5 | 3.17E-01 | ECM regulators |
| LOXL3 | 1.47 | 3.16E-01 | ECM regulators |
| MMP13 | 1.3 | 5.31E-01 | ECM regulators |
| MMP15 | 9.97 | 3.78E-17 | ECM regulators |
| MMP2 | 0.91 | 4.92E-01 | ECM regulators |
| MMP9 | -1.26 | 9.38E-01 | ECM regulators |
| PCOLCE | -0.47 | 9.79E-01 | ECM regulators |
| PLOD3 | 0.26 | 7.54E-01 | ECM regulators |
| TGM2 | -0.08 | 8.90E-01 | ECM regulators |
| TIMP2 | -0.8 | 9.54E-01 | ECM regulators |
| TIMP3 | 2.67 | 2.09E-02 | ECM regulators |
| ANXA2 | 0.08 | 8.57E-01 | ECM-affiliated proteins |
| COLEC12 | 0.38 | 7.42E-01 | ECM-affiliated proteins |
| CTHRC1 | 2.36 | 2.10E-01 | ECM-affiliated proteins |
| HAPLN3 | 0.7 | 6.44E-01 | ECM-affiliated proteins |
| NID1 | -0.05 | 8.83E-01 | ECM-affiliated proteins |
| NID2 | 0.79 | 6.88E-01 | ECM-affiliated proteins |
| ADIPOQ | -5.2 | 8.93E-03 | Secreted factors |
| AHSG | 9.97 | 3.78E-17 | Secreted factors |
| ANGPTL2 | 2.41 | 1.97E-01 | Secreted factors |
| ANGPTL5 | 3.96 | 3.45E-03 | Secreted factors |
| C1QTNF3 | 2.59 | 1.64E-01 | Secreted factors |
| C1QTNF5 | 3.63 | 9.31E-03 | Secreted factors |
| CPN2 | -1.29 | 7.86E-01 | Secreted factors |
| FER | -1.7 | 6.34E-01 | Secreted factors |
| FGL1 | 0.18 | 7.74E-01 | Secreted factors |
| MGP | 9.97 | 3.78E-17 | Secreted factors |
| TGFB1 | 2.94 | 1.05E-01 | Secreted factors |
| TGFB2 | 1.92 | 1.13E-01 | Secreted factors |
| LRRC15 | 3.66 | 3.71E-02 | Other |
| PGM5 | 0.12 | 8.53E-01 | Other |
| PTPRZ1 | 9.97 | 3.78E-17 | Other |
| SGCD | 9.97 | 3.78E-17 | Other |
| SNTB1 | -0.07 | 8.90E-01 | Other |
| SNTB2 | 0.26 | 7.50E-01 | Other |
| SRPX2 | 0.31 | 7.40E-01 | Other |


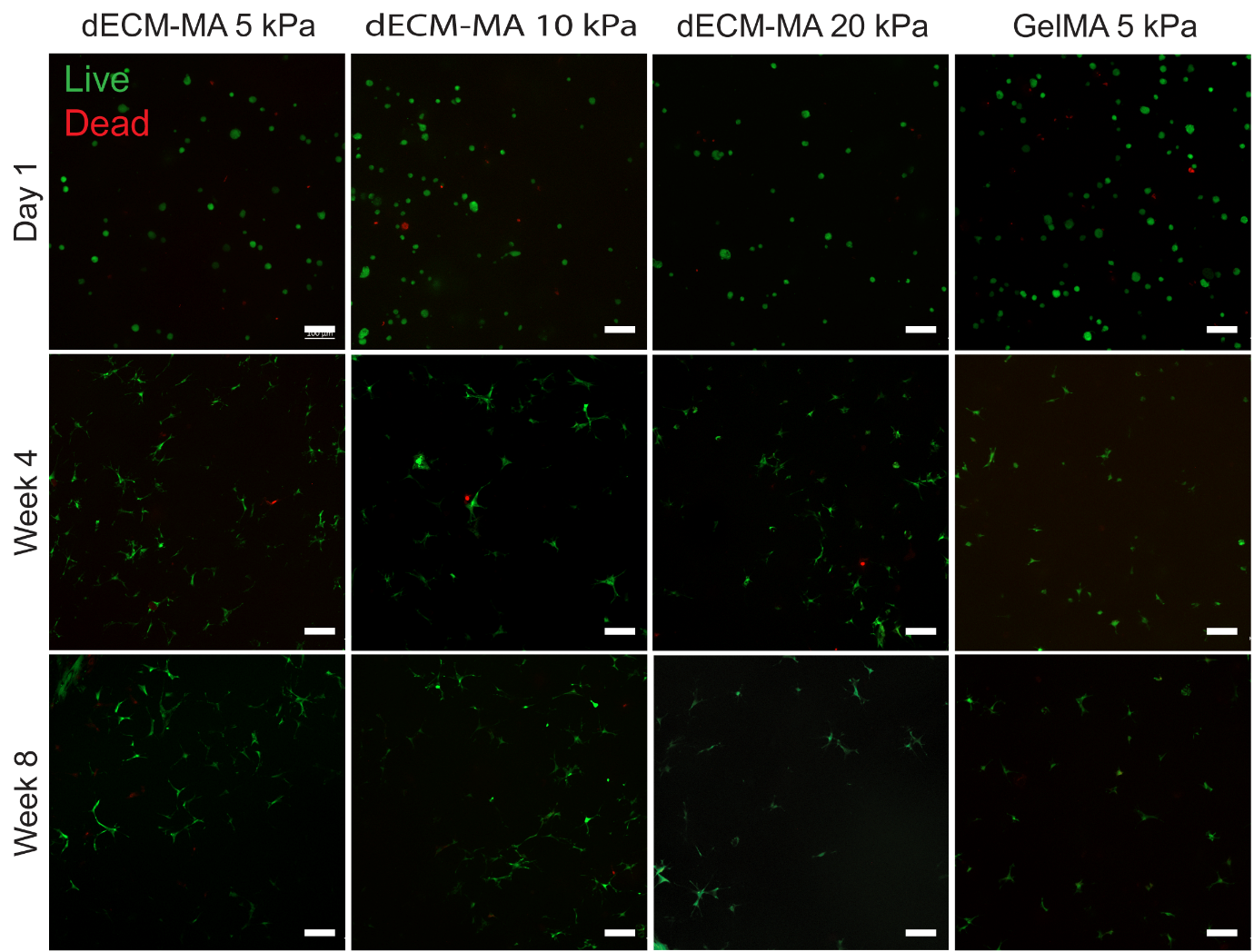


**Figure S5.** Cell viability of the dECM-MA gels decellularized with 3.4 M NaCl compared to GelMA. epresentative images Live/Dead staining using FDA (green, live cells) and in PI (red, dead cells) of the hOBs encapsulated in dECM-MA decellularized with NaCl and GelMA using 2x10^6^ cells.ml^-1^ on day 1, week 4 and 8. Scalebar = 100 µm.


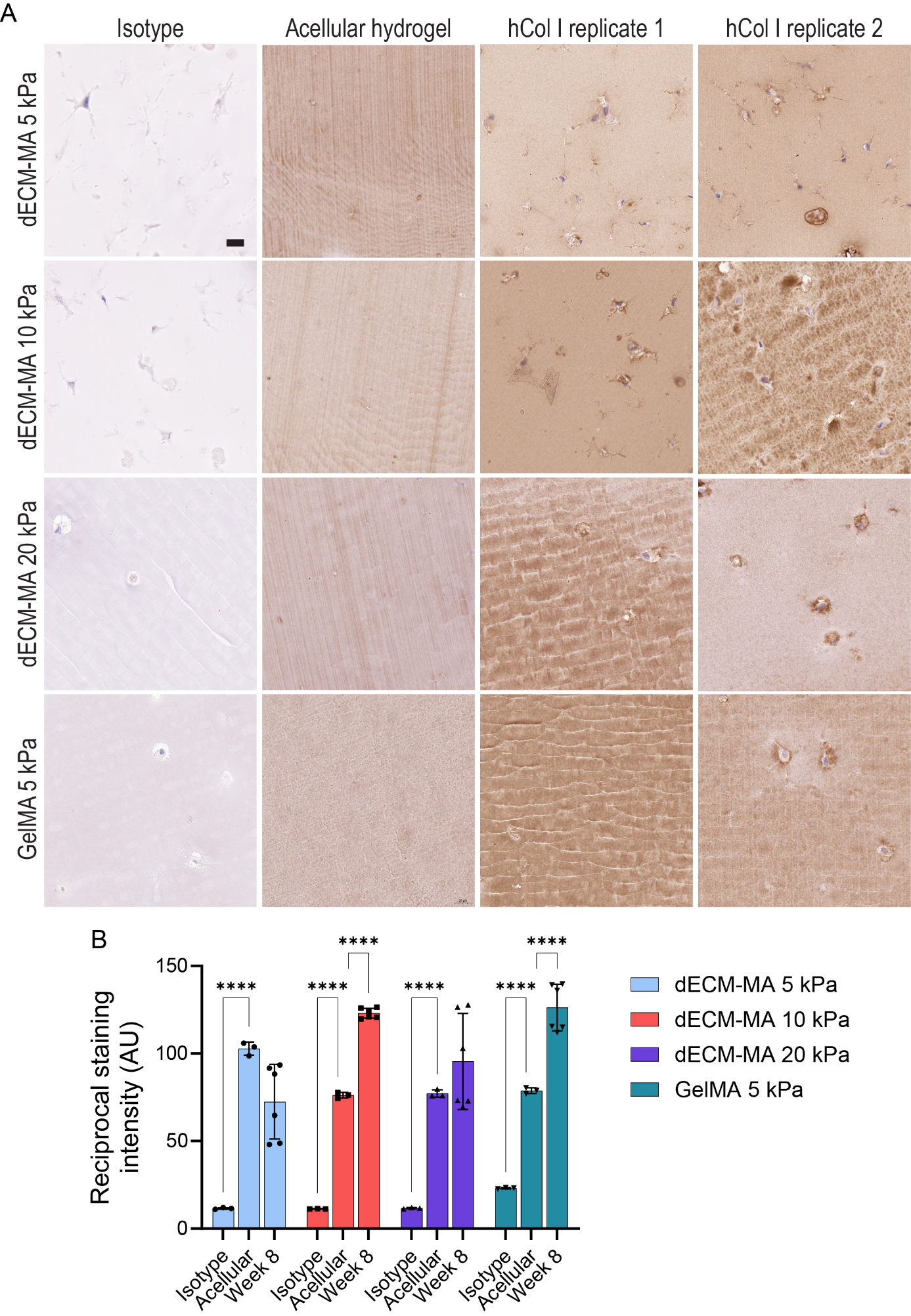


**Figure S6.** Immunohistochemistry analysis of the dECM-MA and GelMA hydrogels. A) Collagen 1 staining on 4 µm sections using 40x magnification for the isotype (only secondary antibody), acellular hydrogels and 2 biological replicates. The black scalebar = 20 µm. B) Reciprocal staining intensity of DAB staining (RSI = 255 – mean gray value) of the collagen 1 staining of 3 ROIs of 2 biological replicates. (n = 6, 2 biological replicates, 3 technical replicates). Statistical analysis was performed using a two-way ANOVA, with significant findings indicated by **** (p < 0.0001).


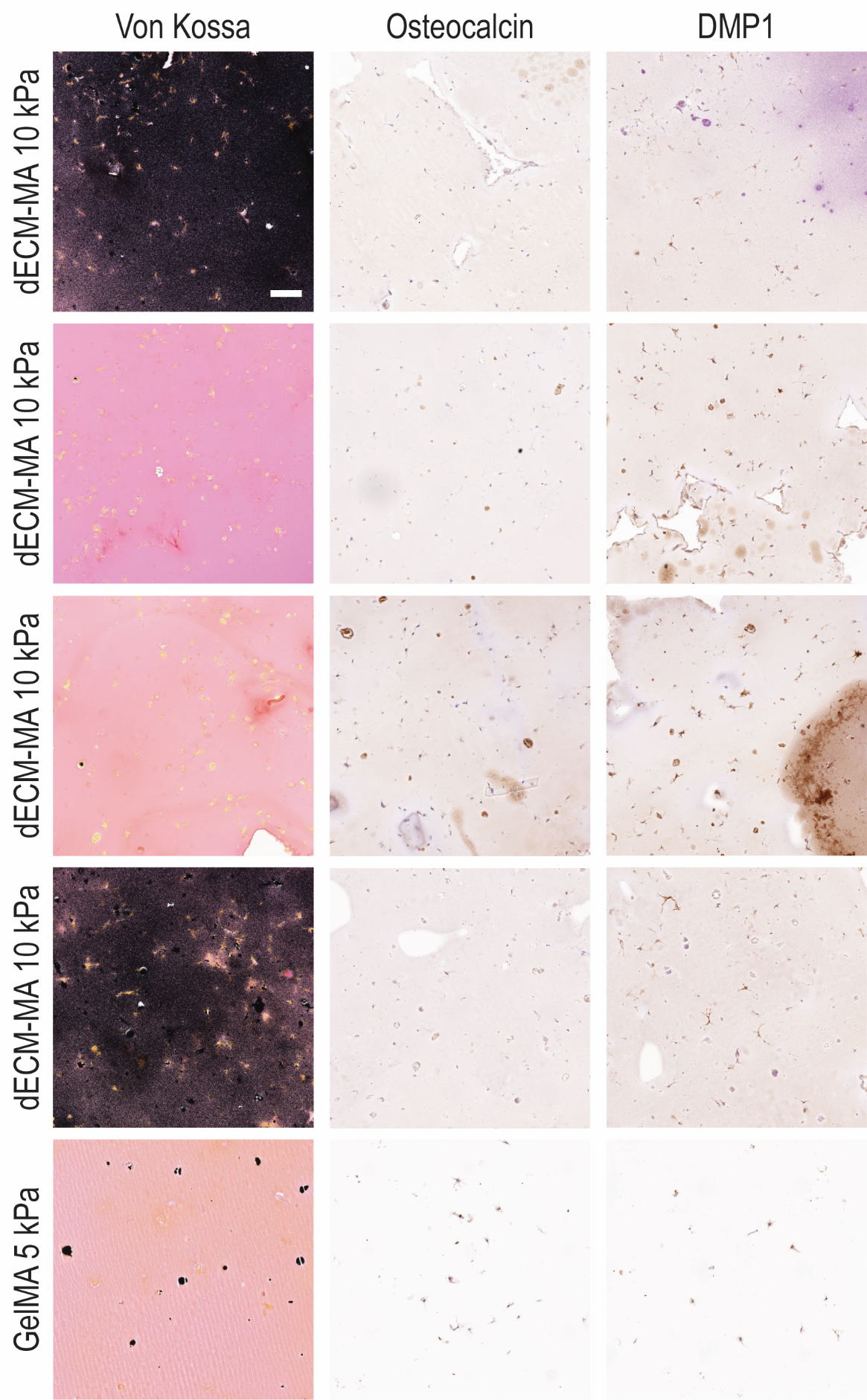


**Figure S8.** Immunohistochemistry analysis of the dECM-MA and GelMA hydrogels. Von Kossa, Osteocalcin and DMP-1 staining on 4 µm sections using 40x magnification for 4 dECM-MA 10 kPa biological replicates and a representative GelMA 5 kPa representative. The black scalebar = 100 µm.
